# Transcriptional coregualtor NUPR1 maintains tamoxifen resistance in breast cancer cells

**DOI:** 10.1038/s41419-021-03442-z

**Published:** 2021-02-04

**Authors:** Lingling Wang, Jiashen Sun, Yueyuan Yin, Yanan Sun, Jinyi Ma, Ruimin Zhou, Xinzhong Chang, Ding Li, Zhi Yao, Shanshan Tian, Kai Zhang, Zhe Liu, Zhenyi Ma

**Affiliations:** 1 Tianjin Key Laboratory of Medical Epigenetics, Key Laboratory of Breast Cancer Prevention and Therapy (Ministry of Education), Key Laboratory of Immune Microenvironment and Disease (Ministry of Education), Department of Immunology, Biochemistry and Molecular Biology, Tianjin Medical University, Tianjin, China; 2 Department of Breast Cancer, Breast Cancer Center, Tianjin Medical University Cancer Institute and Hospital, Tianjin, China; 3 Department of Clinical Laboratory, National Clinical Research Center of Cancer, Key Laboratory of Cancer Prevention and Therapy, Tianjin Medical University Cancer Institute and Hospital, Tianjin, China

**Keywords:** Macroautophagy, Breast cancer

## Abstract

To support cellular homeostasis and mitigate chemotherapeutic stress, cancer cells must gain a series of adaptive intracellular processes. Here we identify that NUPR1, a tamoxifen (Tam)-induced transcriptional coregulator, is necessary for the maintenance of Tam resistance through physical interaction with ESR1 in breast cancers. Mechanistically, NUPR1 binds to the promoter regions of several genes involved in autophagy process and drug resistance such as *BECN1*, *GREB1*, *RAB31*, *PGR*, *CYP1B1*, and regulates their transcription. In Tam-resistant ESR1 breast cancer cells, *NUPR1* depletion results in premature senescence in vitro and tumor suppression in vivo. Moreover, enforced-autophagic flux augments cytoplasmic vacuolization in *NUPR1*-depleted Tam resistant cells, which facilitates the transition from autophagic survival to premature senescence. Collectively, these findings suggest a critical role for NUPR1 as a transcriptional coregulator in enabling endocrine persistence of breast cancers, thus providing a vulnerable diagnostic and/or therapeutic target for endocrine resistance.

## Introduction

Macroautophagy (hereafter autophagy) is a cellular self-degradation process that captures superfluous components for lysosomal clearance to improve survival during stress^1^. Pioneering studies documented that the primary roles of autophagy are the maintaining of cellular homeostasis, unconventional secretion, plasma membrane repair, and cellular differentiation and development^2^. However, both deficient autophagy and excessive autophagy are associated with human diseases, including cancers^3^. Therefore, the role of autophagy in cancer cells is viewed as controversial, as interventions to both enhance and inhibit autophagy have been proposed as cancer therapies under context-dependent conditions^4^. In addition, the precise molecular mechanisms of aberrant autophagy regulation remain unclear, highlighting the contextual role of autophagy in cellular demise in cancer^5^. For example, cancer cells evade apoptotic pathways to facilitate their growth in the present of drug treatments, which often results in drug persistence and/or drug resistance to therapy and tumor recurrence^6^. Thus, alternative approaches beyond apoptosis induction are needed to eradicate drug-resistant cancer cells.

Transcriptional regulator nuclear protein 1 (NUPR1, also called p8 or candidate of metastasis 1, Com-1) is a disordered protein and its upregulation is highly associated with malignant characteristics of cancer as well as with chemoresistance^7–10^. Recent studies suggest more diverse roles for its functional relevance in migration, invasion and autophagy regulation, indicating its broad regulatory control of tumorigenesis^11–13^. For example, NUPR1 activates Runt-related transcription factor 2 (RUNX2) and the NUPR1/RELB/IER3/RUNX2 pathway plays a pivotal role in hepatocarcinogenesis^11^. NUPR1 is also regulated by matrix rigidity via the Yes-associated protein (YAP)-dependent Hippo signaling pathway^12^. Indeed, Food and Drug Administration (FDA)-approved chemicals such as trifluoperazine dihydrochloride or its derived compounds bind NUPR1 and inhibit tumorigenesis in human pancreatic ductal adenocarcinoma-derived xenografts in nude mice^14^. Mechanistically, NUPR1 binds the Polycomb protein RING1B and is involved in carcinogenesis and chemoresistance through chromatin remodeling or non-coding RNA regulation^15,16^. However, the downstream mechanisms of NUPR1 in the context of cancer and whether these mechanisms are specific to cell types, or shared with other regulatory interventions remain largely unknown.

Most cases of breast cancer are estrogen receptor alpha (ERα) positive and ER antagonist, such as tamoxifen (Tam) and its derivatives are clinical antibreast cancer drugs^17^. However, endocrine therapy develops resistance and attempts to administer therapeutic interventions against chemoresistance have proven to be challenging^18^. The potential pharmacogenetic explanations for Tam resistance involve ER-regulatory signaling network connecting hormonal endocrine therapy are controversial, leaving it an open question for further investigation^18^. Indeed, it has been noticed that cancer cells by cytotoxic agents treatment durably induces premature senescence with states of proliferative arrest, potentiating the implications of modulated senescence for the outcome of cancer intervention^19,20^. We postulated that impaired autolysosomal clearance, which is needed to protect cancer cells from a variety of stresses to which they are highly susceptible, could be responsible for enhancing malignant progression. Here, we investigate how NUPR1 accelerates and maintains the development of Tam-induced resistance in breast cancer cells, and what this means in relation to enhancing antiestrogen therapy.

## Results

### *NUPR1* is induced by Tam treatment and correlates with lower overall survival rate

**Fig. 1:**
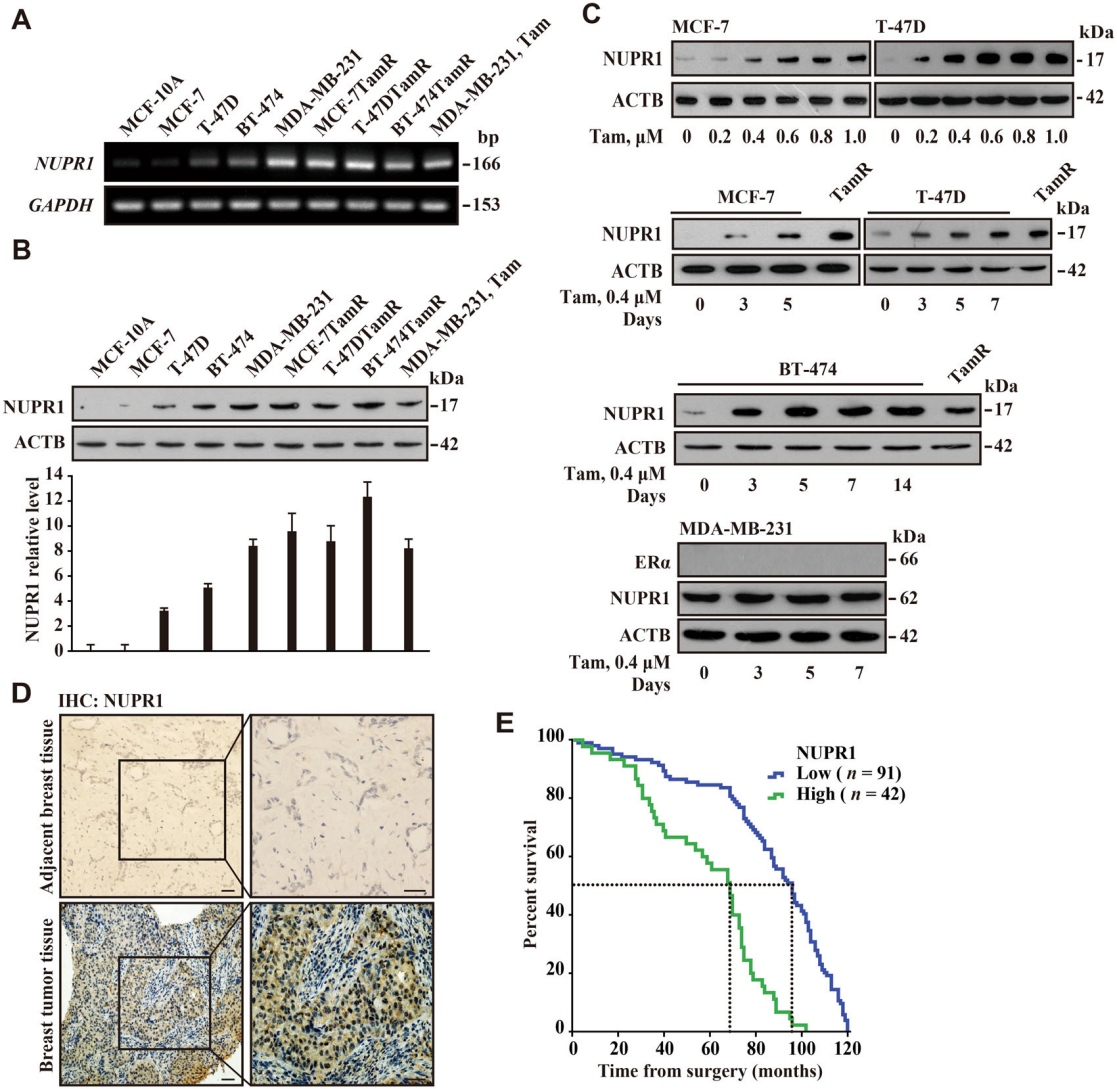
NUPR1 is induced by Tam and correlates with lower overall survival. **A** To determine *NUPR1* expression levels in breast cancer cell lines as indicated, RT-PCR products were evaluated by agarose gel electrophoresis. RNA levels were normalized to *GAPDH* expression. bp base pairs. **B** Immunoblots were carried out with anti-NUPR1 and anti-ACTB antibodies in the cell lines used in **A**. Experiments in both **A** and **B** were performed in triplicate, yielding similar results. Lower panel, NUPR1 relative protein level. **C** Endogenous NUPR1 was induced by 0.4 µM Tam in ESR1-positive breast cancer cell lines as indicated. Western blot analysis of these cells treated for the indicated time was carried out as described in **B**. **D** Representative distribution of NUPR1 by IHC in clinical breast cancer specimens and their adjacent noncancerous tissues from the patient of origin (IHC, brown). Scale bars, 50 μm. **E** Kaplan–Meier plots of overall survival after surgery for 133 breast cancer subjects with low (0–5.0 staining scores, blue lines; *n* = 91) or high (5.1–10.0 staining scores, green lines; *n* = 42) NUPR1 expression (*P* = 0.0021). The hazard ratio for high NUPR1 expression was 6.998 (CI 4.128–11.862, log-rank test). Median survival was more than 95.4 months for the low NUPR1 expression group versus 68.1 months for the high NUPR1 expression group.

Since autophagy is closely connected to drug resistance^21^, we initially asked whether NUPR1 expression is differentially involved during the development of Tam resistance in breast cancer cells. First, we individually generated tamoxifen-resistant (TamR) clones derived from three estrogen receptor alpha (ESR1)-positive breast cancer cell lines MCF-7, T-47D, and BT-474 by exposing them to 2 μM Tam for a period of over 15 months. Initially, cell viability rates were dramatically decreased in the estrogen sensitive MCF-7 cells with Tam treatment, compared to the treatment of vehicle (ethanol, OH) (Fig. S1a). After 15 months of Tam treatment, the new-subline cells were used as TamR cells which were maintained in the growth medium containing 0.4 μM Tam. RT-PCR analysis showed that the mRNA level of *NUPR1* was significantly higher in TamR cells than that in control cells (Fig. 1A). In agreement with the RT-PCR data, the protein levels of NUPR1 were also clearly elevated in MCF-7TamR, T-47DTamR, and BT-474TamR cells, compared to MCF-10A mammary epithelial cells with little or low NUPR1 expression (Fig. 1B, C). In contrast, Tam treatment had no significant effect on NUPR1 protein level in MDA-MB-231 cells (Fig. 1A–C), which are Tam resistant cells^22^. Moreover, Tam treatment induced NUPR1 expression at a consistent level in a time-dependent and dose-dependent manner in three ERα-positive breast cancer cell lines, which are TamR after 15 months of treatment (Fig. 1C). These data suggest that NUPR1 is involved in the development of resistance to Tam.

Next, using a tissue array of primary invasive ductal carcinoma of human breast (*n* = 133), we conducted immunohistochemistry (IHC) and found that NUPR1 was strongly detected in the nucleus in 30% of the breast tumor tissues, compared with that found by negative staining in adjacent noncancerous breast tissues (Fig. 1D). Using immunocytochemistry analysis, we found that *NUPR1*-depleted MCF-7TamR cells exhibited decreased NUPR1 staining, supporting antibody specificity (Fig. S1b). Moreover, the NUPR1 protein level was significantly associated with survival time after surgery (Fig. 1E and Table S1). Of 133 subjects of breast cancer tissues, medium survivals were 95.4 months with low NUPR1 staining score (*n* = 91) and 68.1 months with high NUPR1 scores (*n* = 42, *P* = 0.0021, Fig. 1E). High NUPR1 protein level was a strong predictor of low survival rates in breast cancer patients (hazard ratio 6.998, CI 4.128–11.862, Fig. 1E). NUPR1 staining scores did not significantly correlate with age, TNM, or hormone status (Table S1). Collectively, these data indicate that high expression level of NUPR1 correlates with low overall survival rates.

### *NUPR1* depletion induces premature senescence through impaired autolysosomal process

**Fig. 2:**
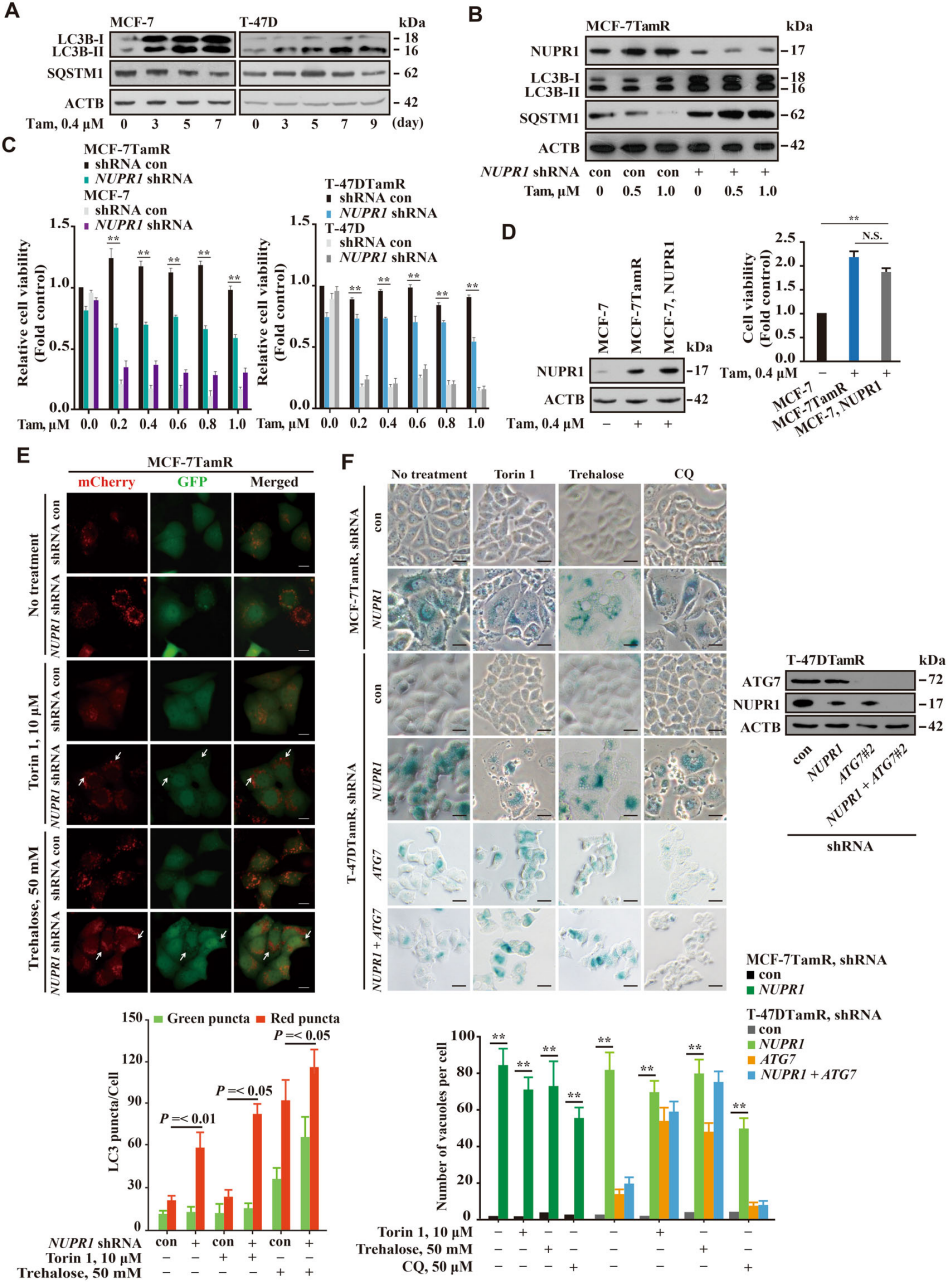
NUPR1 mediates autophagic survival and *NUPR1* depletion induces premature senescence. **A** Immunoblots of LC3B and SQSTM1 were carried out in MCF-7 and T-47D cells treated with 0.4 µM Tam for the indicated time; ACTB served as a loading control (*n* = 3). **B** MCF-7TamR cells were transfected with *NUPR1* shRNA and treated with 0.4 µM Tam. Immunoblots of NUPR1, LC3B, and SQSTM1 were performed as described in **A**. **C** Cellular viability was assessed in *NUPR1*-shRNA MCF-7TamR and T-47DTamR cells treated with Tam for 48 h as well as in the parental control cells. Experiments in **C** were replicated twice, yielding similar results. **P < 0.01, compared to the shRNA control cells. **D** Immunoblot of NUPR1 in MCF-7TamR cells, MCF-7TamR cells transfected with *NUPR1* cDNA (NM_012385) in an expression vector and their corresponding parental cells; ACTB served as a loading control (left panel). Right panel, cell viability assay as described in **C**. N.S. not significant. **E** Analysis of GFP-mCherry-LC3 fluorescent signals. MCF-7TamR cells were transfected with GFP-mCherry-LC3 plasmid, and treated with 10 µm torin 1 or 50 mM trehalose for 10 h. White arrows indicate dilated vacuoles. Lower panel, quantification of the number of GFP or mCherry puncta per cell in *NUPR1*-depleted and control cells (ten cells per group). Error bars represent the mean ± SD. **F** Representative light microscopy images of GLB1 staining in MCF-7TamR and T-47DTamR cells with *NUPR1* and/or *ATG7* shRNA following the indicated treatment for 10 h. Lower panel, quantification of the dilated vacuoles from three independent experiments (mean ± SD, *n* = 10). Right panel, immunoblots confirmed the knockdown efficiency of shRNAs against human *NUPR1* and/or *ATG7*, with fire fly luciferase as a negative control (con) and ACTB as an internal control. **P < 0.01, compared to the shRNA control cells.

Cancer cells rely on upregulated autophagy to survive intrinsic and/or extrinsic stress and to enhance growth and aggressiveness^6^. We then investigated whether NUPR1 was involved in the autophagy process during the development of Tam resistance. Upon treatment with 0.4 μM Tam MCF-7 and T-47D cells showed an increased conversion of LC3B-I to LC3B-II, and a decreased accumulation of SQSTM1 (sequestosome 1)/p62 after 7 days of Tam treatment, indicating enhanced autolysosomal clearance (Fig. 2A). To further confirm this observation, we depleted *ATG5* or *ATG7* using shRNA in both parental and TamR cells, respectively (Fig. S2a, left panels). ATG5 and ATG7 play key roles in the elongation of autophagophore^1^. We found that depletion of either *ATG5* or *ATG7* significantly restrained autophagy process compared with their respective controls (Fig. S2a). Likewise, ultrastructural analysis under a transmission electron microscope indicated a significant increase in the number of swelled cytoplasmic vacuoles in *NUPR1*-depleted MCF-7TamR cells compared with that in knockdown control cells (Fig. S2b).

To determine the relevance of NUPR1 in autophagy-mediated Tam resistance, we depleted endogenous *NUPR1* in MCF-7TamR and T-47DTamR cells using shRNA and monitored autophagic flux change. Infection of three different shRNA sequences against *NUPR1* resulted in a strong depletion of *NUPR1* expression compared to that in cells infected with the firefly luciferase control (control shRNA, con) (Fig. 2B, left). *NUPR1*-depleted MCF-7TamR cells showed more LC3B-I to LC3B-II conversion and SQSTM1 accumulation, resulting in an impaired autophagic process (Fig. 2B, right). Moreover, *NUPR1* depletion resulted in an increased sensitivity of MCF-7TamR and T-47DTamR cells to the antiproliferative effects of Tam, compared with their parental control cells (Fig. 2C). In line with this observation, *NUPR1* overexpression resulted in a protective effect against Tam treatment (Fig. 2D). In ESR1-negative MDA-MB-231 cells, autophagy inducer (torin 1 or trehalose) treatments showed a reduced autophagic flux upon *NUPR1* depletion (Fig. S2c). Thus, these data indicate that NUPR1 maintains autophagy flux in Tam resistant cells, which may benefit cellular survival.

To determine the mechanism in this process, we transfected a tandem-tagged mCherry-GFP-LC3B (hereafter referred to as mCherry-GFP-LC3) plasmid into *NUPR1*-depleted MCF-7TamR cells to monitor the localization of LC3 puncta. In mCherry-GFP-LC3 MCF-7TamR cells, *NUPR1* depletion dramatically increased the number of mCherry-positive, GFP-negative autolysosomes (red puncta) (Fig. 2E). Previously, we noticed that *NUPR1* depletion results in cytoplasmic vacuolization and premature senescence in lung cancer cells^13^. Next, we asked whether this effect also occurred in TamR breast cancer cells. Indeed, *NUPR1* depletion in MCF-7TamR or T-47DTamR cells treated with trehalose (an MTOR-independent inducer of autophagy) or torin 1 (an MTOR inhibitor) increased autolysosome formation and cytoplasmic vacuolization (Figs. 2E, F). In contrast, CQ treatment decreased cytoplasmic vacuolization and staining for GLB1, a premature senescence marker in MCF-7TamR and T-47DTamR cells upon *NUPR1* depletion (Fig. 2F). *ATG7* depletion did not inhibit senescence upon *NUPR1* knockdown in T-47DTamR cells (Fig. 2F). Additionally, neither the active forms of lysosomal proteases cathepsin D nor the MTOR pathway were significantly altered in this process (Fig. S2d and S2e), indicating that autolysosomal degradation upon *NUPR1* depletion is independent of the MTOR pathway in TamR breast cancer cells. Finally, we also found that combined treatment with trehalose and Baf A1 did not change LC3B-II and SQSTM1 accumulation upon *NUPR1* depletion (Fig. S2f). Collectively, these data indicate that *NUPR1* depletion renders TamR breast cancer cells more susceptible to premature senescence and cytoplasmic vacuolization.

### *NUPR1* depletion overcomes Tam resistance in vitro and in vivo

**Fig. 3:**
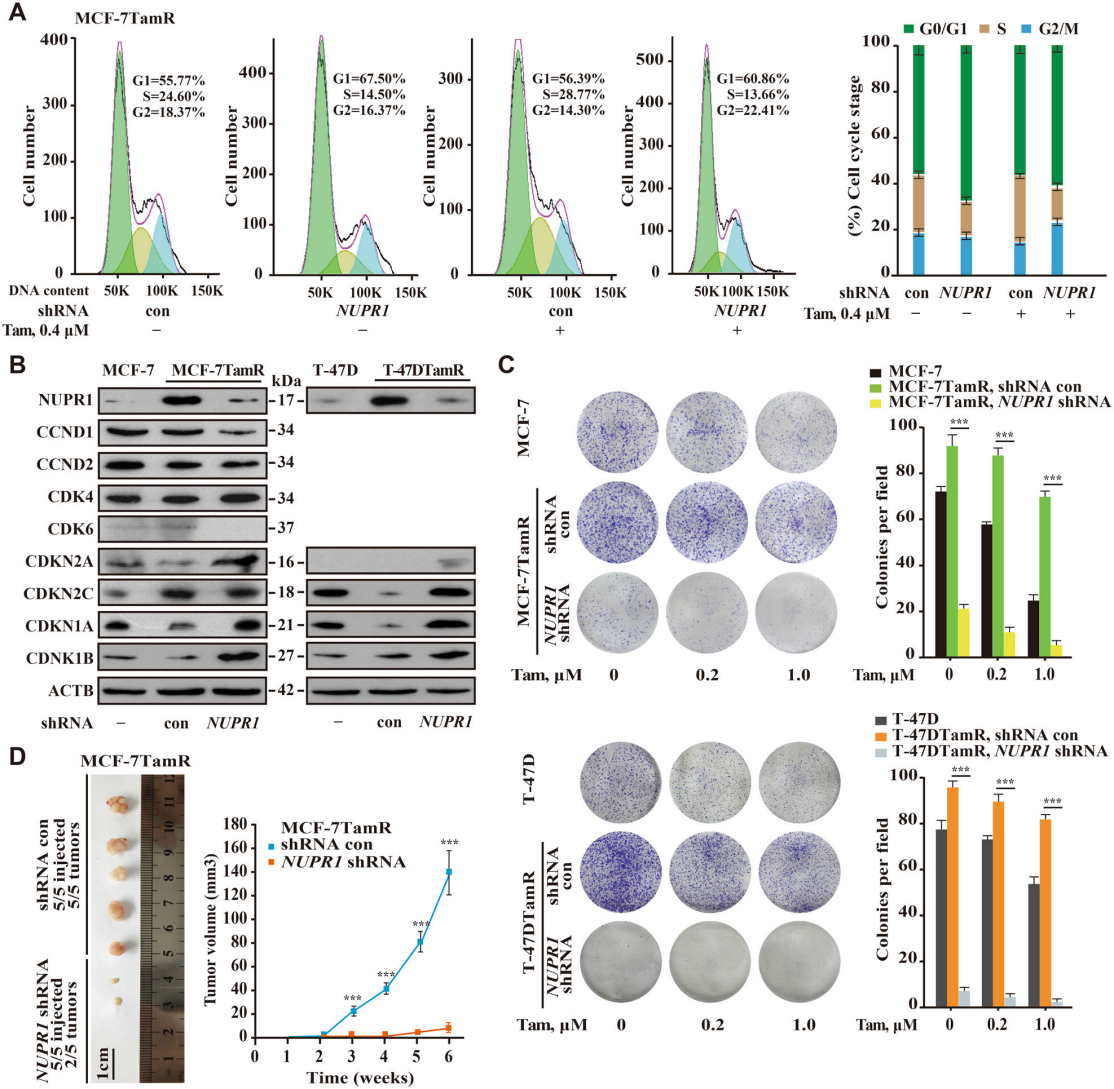
*NUPR1* depletion overcomes Tam resistance in vitro and in vivo. **A** Cell cycle analysis by flow cytometry of *NUPR1*-depleted MCF-7TamR cells, treated with vehicle (ethanol) or 0.4 μM Tam. The percentage of cells in G0/G1, S, and G2/M phases were determined from three independent experiments (right panel, mean ± SD). **B** Immunoblot of the indicated proteins in cells upon *NUPR1* depletion by shRNA, with ACTB as a loading control. **C** Representative images of the clonogenic assay in MCF-7TamR or T-47DTamR cells upon *NUPR1* depletion and their corresponding shRNA con cells. The percentage of colonies is expressed in bar graphs (right panel) as the mean ± SD. of three separate experiments, each performed in triplicate. ***P < 0.001, compared to the shRNA control cells. **D** *NUPR1*-depleted MCF-7TamR cells were orthotopically and bilaterally implanted into the mammary fat pads of female nude mice (*n* = 5). Microphotographs of tumors collected (left panel) at 6 weeks after injection and the tumor growth curve (right panel, mean ± SD) are shown. ***P < 0.001, compared to the shRNA control cells.

Since Tam induces *NUPR1* transcription, *NUPR1* depletion may be detrimental to TamR breast cancer cells. Indeed, *NUPR1* depletion alone was able to induce G0/G1 cell cycle arrest and decrease the percentage of cells in S phase in MCF-7TamR cells, even in the presence of 0.4 μM Tam (Fig. 3A). Notably, the loss of the cyclin-dependent kinase (CDK) inhibitor CDKN1A/p21 (also a senescence-specific marker) could mediate Tam resistance as shown in Fig. 3B, is consistent with a previous report^23^. Additionally, other cell cycle inhibitors such as CDKN2A/p16 and CDKN1B/p27 were also increased upon *NUPR1* depletion in MCF-7TamR and T-47DTamR cells, indicating that NUPR1 is necessary for cell cycle maintenance (Fig. 3B). In these cell lines, Tam treatment alone decreased colony formation in soft agar, and the combination of *NUPR1* shRNA and Tam treatment, when compared with either treatment alone, significantly reduced colony formation (Fig. 3C). Consistent with these analyses, the results of the transwell assay showed that *NUPR1* depletion in these cells decreased the cytokine-dependent invasion phenotype (Fig. S3a). Additionally, *NUPR1* depletion in these TamR cells rather than the parental cells significantly decreased tumor volume, and the number of tumors formed in the mammary fat pad in an athymic nude mouse model with Tam treatment (Fig. 3D and Fig. S3b). Significantly, the histological analysis of the tumors showed that these *NUPR1* knockdown tumors contained fewer proliferating cell nuclear antigen (PCNA)-positive cells (Fig. S3c). Thus, downregulating *NUPR1* decreases tumor size, abrogating the effect of Tam resistance on tumor growth due to the activation of premature senescence. Taken together, these data indicate that *NUPR1* depletion overcomes Tam resistance in breast cancer cells.

### NUPR1 physically interacts with ESR1

**Fig. 4:**
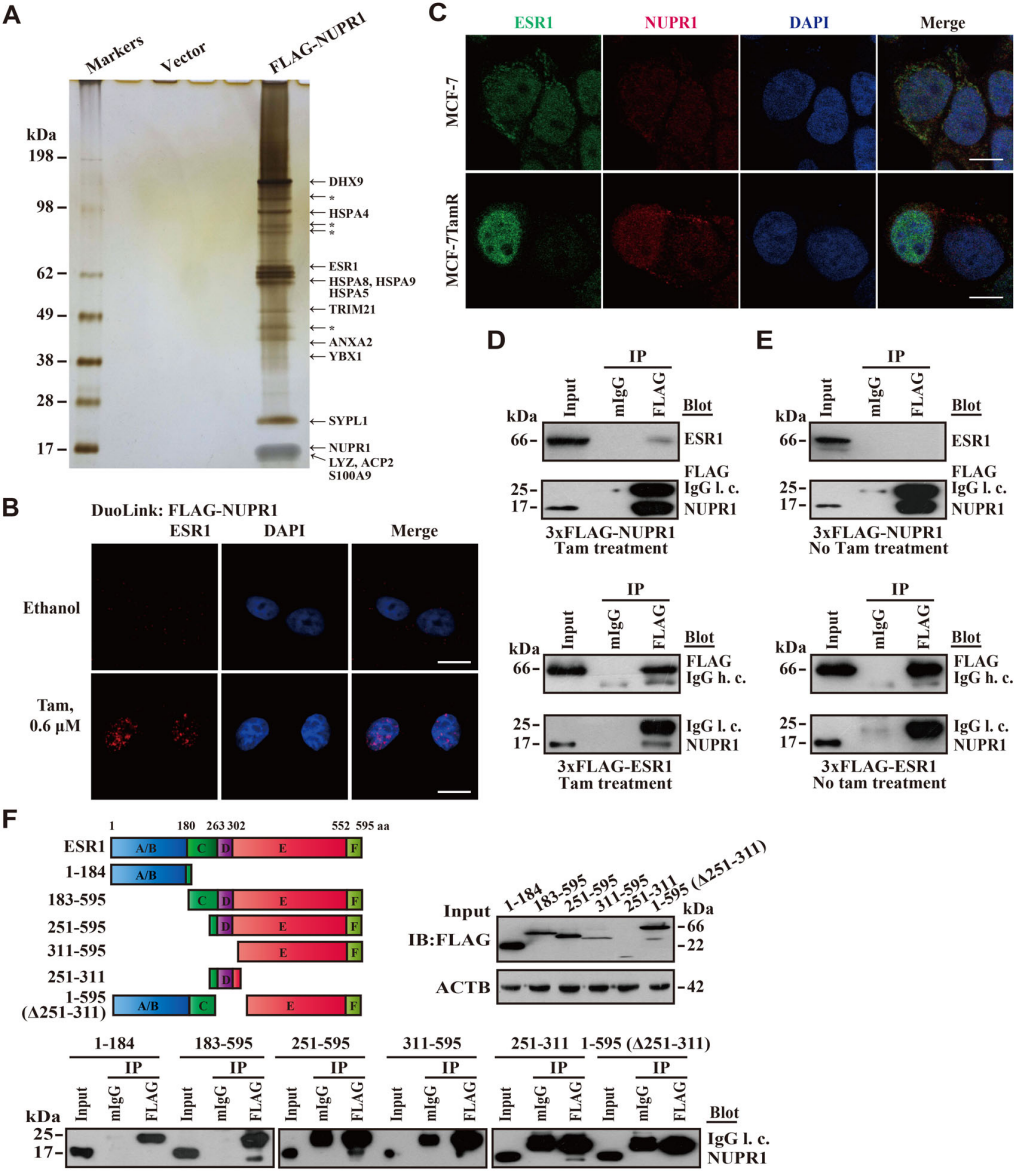
NUPR1 physically interacts with ESR1. **A** Immuno-purification of NUPR1-containing protein complexes. Cellular extracts from MCF-7TamR cells stably expressing FLAG (empty vector, control) or FLAG-NUPR1 were immunopurified with an M2 anti-FLAG affinity gel and eluted with 3× FLAG peptide. The eluates were resolved by SDS-PAGE, and bands of interest were analyzed by mass spectrometry. *, nonspecific binding proteins. **B** DuoLink assay of the interaction between FLAG-tagged NUPR1 and endogenous ESR1 (red) in MCF-7TamR cells treated with vehicle (ethanol) or 0.6 μM Tam for 24 h. Nuclei were counterstained with DAPI (blue). Scale bar, 10 μm. **C** Representative immunofluorescence (IF) images of endogenous NUPR1 and ESR1 colocalization in MCF-7TamR but not in their parental cells. Green, ESR1; Red, NUPR1. Nuclei were counterstained with DAPI (blue). Scale bar, 10 μm. **D**, **E** Flag-tagged *NUPR1* or *ESR1* was transfected into MCF-7TamR cells with or without Tam treatment for 48 h. Immunoprecipitation (IP) of whole-cell lysates with an M2 anti-FLAG antibody and Western blotting analysis of the indicated proteins were conducted. IgG h. c. IgG heavy chain, IgG l.c. IgG light chain. **F** Co-IP assay of NUPR1 and ESR1. FLAG-tagged ESR1 or its serial deletions was transfected into MCF-7TamR cells and Western blotting analysis of the indicated proteins were conducted as in **D**, with ACTB as a loading control. Left: schematic representation of full-length ESR1 protein and its truncated forms. A/B, activating function; C, DNA-binding domain; D, nuclear localization signal; E and F, hormone-binding domain.

To better understand the mechanistic role of NUPR1 during Tam resistance, we employed affinity purification and mass spectrometry to identify proteins that associated with FLAG-tagged NUPR1 in MCF-7TamR cells. NUPR1 was found in a complex with ANXA2, TRIM21, YBX1, S100A9, HSPA8, HSPA9, HSPA4, HSPA5, and ESR1 (Fig. 4A and Table S4), suggesting that these proteins might play a role in its functional regulation. One of the identified NUPR1 binding partners was ESR1, a transcription factor that mediates endocrine resistance by recruiting transcriptional coactivators or corepressors to the regulatory regions of its target genes^24^. To confirm the interaction between NUPR1 and ESR1, we used three different detection methods: the proximity ligation assay (PLA), immunofluorescence staining and the coimmunoprecipitation (co-IP) assay. We found that colocalization was predominantly enriched in the nucleus but less pronounced in the cytosol using PLA and immunostaining, respectively (Fig. 4B, C). Interestingly, the association of NUPR1 and ESR1 in MCF-7TamR cells was demonstrated by co-IP with Tam treatment, compared with no Tam treatment control (Fig. 4D, E), consistent with the PLA data (Fig. 4B, lower panel). In addition, the presence of ESR1 in the NUPR1 complex was further confirmed by the truncated ESR1 deletion assay, indicating that the nuclear localization signal (NLS) (251–311 aa) of ESR1 was necessary to bind NUPR1 (Fig. 4F). Collectively, these data suggest that a physical interaction exists between NUPR1 and ESR1.

### NUPR1 and ESR1 coordinately regulate transcription profile

**Fig. 5:**
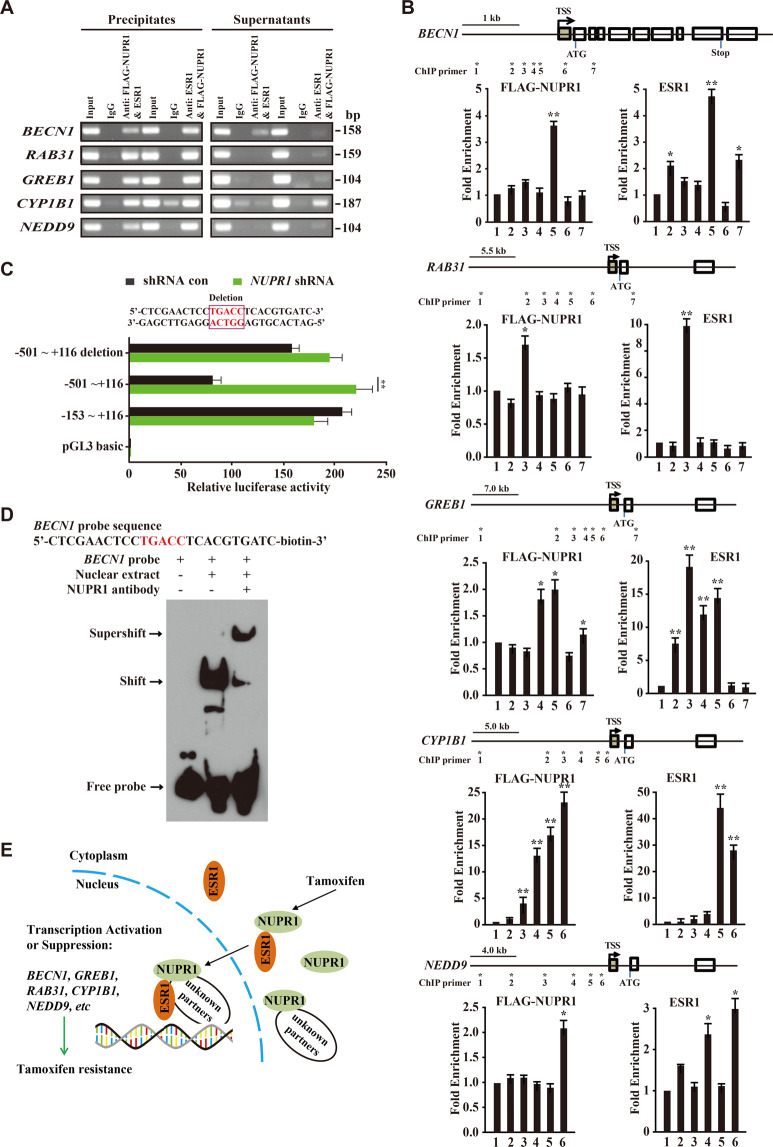
NUPR1 directly suppresses *BECN1* transcription in MCF-7TamR cells. **A** ChIP-re-ChIP showing the enhanced occupancy of FLAG-NUPR1 and ESR1 on the promoters of the indicated genes. Sequential antibodies used for the first ChIP and second ChIP are indicated above the lanes. **B** Quantitative RT-PCR was performed to confirm transcriptional changes of the indicated genes identified from the ChIP-seq data. RNA levels are presented as the relative fold change compared to the levels in the control shRNA samples. The mean ± SD of three replicates is shown (*n* = 3). **C** Diagram shows the deletion of the luciferase reporter upstream of the *BECN1* TSS (upper panel); luciferase reporter studies with a mutation of the ESR1 binding site within the −501 to +116 segment, which partially abrogated basal promoter enhancement (lower panel). The mean ± SD of three independent luciferase reporter activities is shown. **d** EMSA shows the mobility shift of the probe with *BECN1* sequence containing an ESR1 binding site, with a supershift following anti-NUPR1 antibody treatment. **E** Proposed working model for the role of NUPR1:ESR1 in the transcriptional regulation of their targets.

Next, we asked how NUPR1 mediates Tam resistance by interacting with the ESR1-involved transcriptional complex. To this purpose, we conducted the ChIP-re-ChIP assay and found concurrent occupancy of NUPR1 and ESR1 at the promoters of the indicated genes, including *BECN1*, *RAB31*, *GREB1*, *CYP1B1*, and *NEDD9* (Fig. 5A). Furthermore, the occupancy of NUPR1 or ESR1 at these genes’ promoters was also verified by ChIP assays (Fig. 5B). Among these regulated genes, *BECN1* was selected for validation because it is a key player mediating autophagic initiation and regulation, which suggests its function as a tumor suppressor^25^. Indeed, the results of the luciferase reporter assay showed that *NUPR1* knockdown or deletion of the TGACC sequence in the region of −501 to +116 increased *BECN1* promoter activity (Fig. 5C). EMSA also showed that the oligos containing TGACC were bound by NUPR1 antibody (Fig. 5D), indicating that *BECN1* is directly regulated by NUPR1. These observations suggest that ESR1 and NUPR1 cooperatively modulate *BECN1* transcription during Tam resistance. Finally, using the *NUPR1 sgRNA*/CRISPR-dCas9-KRAB system, we successfully repressed NUPR1 (Fig. S4a), resulting in GLB1 activation in MCF-7TamR and T-47DTamR cells (Fig. S4b). Based on these data, we propose a model regarding the role of NUPR1-mediated transcription regulation as shown in Fig. 5E. In endocrine therapy resistant breast cancer cells, ESR1 associates with the transcriptional regulator NUPR1 and regulates the transcription of their targets, resulting in enhanced autophagic survival and more malignant behavior. Therefore, disrupting the interaction between NUPR1 and ESR1 may inhibit endocrine therapy resistance progression.

### A NUPR1-regulated gene signature is both prognostic and predictive for endocrine resistance

**Fig. 6:**
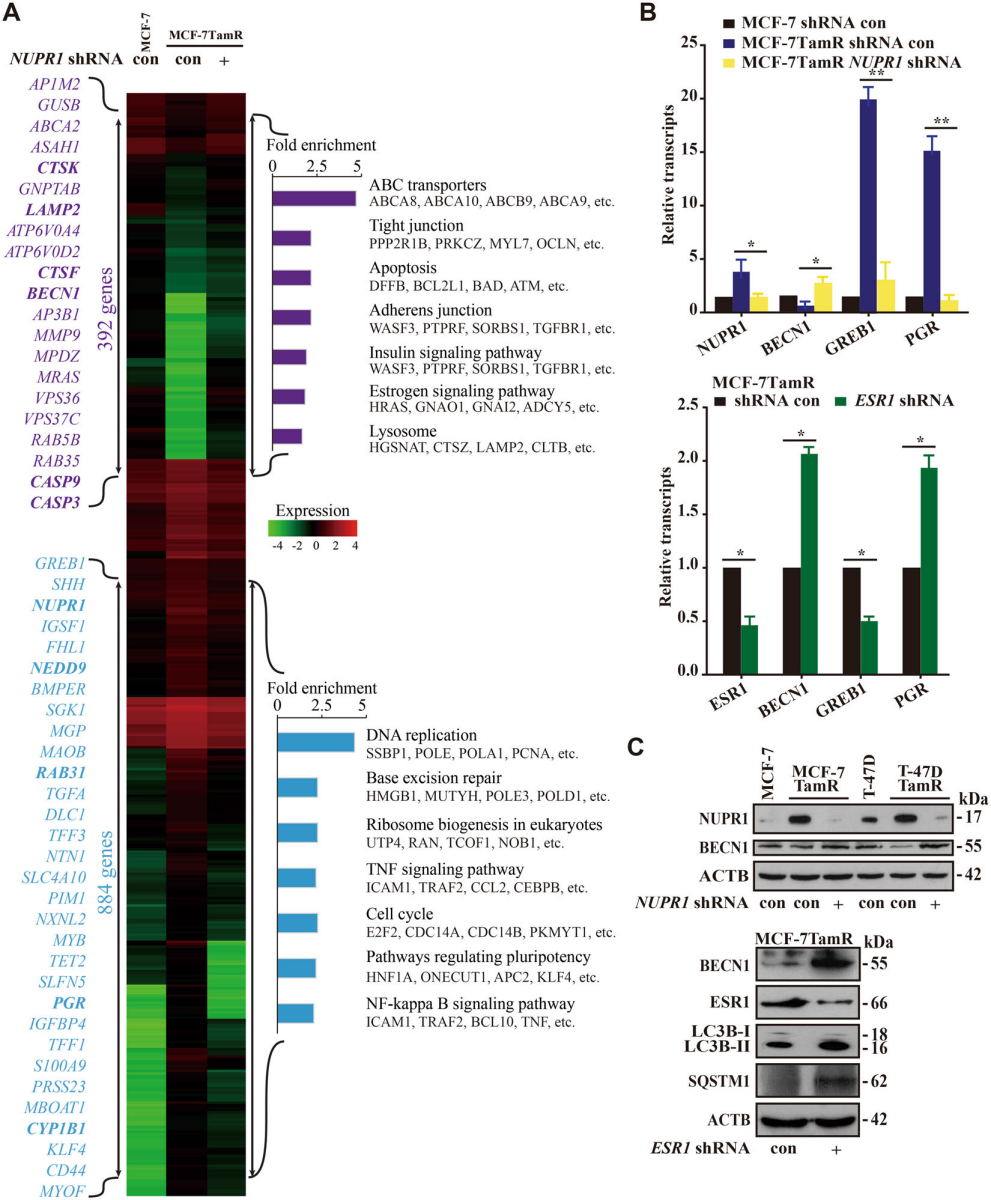
A NUPR1-regulated gene signature is both prognostic and predictive for breast cancer. **A** Functional profiling of genes differentially expressed between MCF-7TamR *NUPR1*-depleted and MCF-7TamR shRNA con cells as well as the parental MCF-7 cells. Representative upregulated (purple) and downregulated genes (light blue) upon *NUPR1* depletion are listed vertically (left) and under each molecular pathway (right). **B** The transcriptional levels of *BECN1*, *GREB1*, and *PGR* were confirmed by quantitative RT-PCR. The expression is shown as fold differences relative to *GAPDH* expression, compared to the levels in shRNA con samples. The mean ± SD of three replicates is shown (*n* = 3). **C** Immunoblot of BECN1, NUPR1, LC3B, SQSTM1 and ESR1 in *NUPR1*- or *ESR1*-depleted cells as indicated, with ACTB as a loading control.

Lastly, to further elucidate the role of NUPR1 in breast cancer, we assessed the transcriptional profile of the affected genes upon *NUPR1* depletion using RNA sequencing. RNA sequencing (RNA-seq) analysis revealed that 1276 NUPR1-dependent genes were differentially altered among MCF-7, MCF-7TamR, and NUPR1-depleted MCF-7TamR transcriptomes (Fig. 6A, and GSE104050). The KEGG pathway analysis (http://www.kegg.jp/kegg/pathway.html) showed the enrichment of genes upon *NUPR1* depletion associated with metabolic pathways, estrogen signaling pathway, cell cycle and the lysosomal process, highlighting the transcriptional flexibility of Tam resistance. Notably, autolysosome-related genes as well as drug resistance genes were downregulated as assessed by real-time quantitative RT-PCR analysis in MCF-7TamR and T-47DTamR cells upon *NUPR1* depletion (Fig. S5a and S5b). Among the top hits, the *PGR* (progesterone receptor), *GREB1* (growth regulation by estrogen in breast cancer 1), and *BECN1* mRNAs were validated (Fig. 6B). Moreover, *ESR1* depletion using lentiviral shRNA increased BECN1 protein levels (Fig. 6C), consistent with the accumulation of LC3B and SQSTM1 by *NUPR1* depletion. Interestingly, we also found that *NUPR1* depletion in MCF-7TamR cells resulted in decreased pro-migratory cytokine interleukin-6 (IL-6) production (Fig. S5c), suggesting a role for NUPR1 in the induction of IL-6 expression in Tam resistant breast cancer cells. These data are consistent with the hypothesis that NUPR1 mediates autophagic survival in endocrine therapy resistance, presumably through NUPR1-mediated transcriptional control.

## Discussion

Despite advances in antiestrogen therapy for patients with ESR1-positive breast cancer, advanced-stage breast cancer remains largely incurable due to therapeutic endocrine therapy resistance and recurrence^26^. Here, we demonstrate that the transcriptional coregulator NUPR1 co-opts ESR1 to mediate Tam resistance through autophagic survival in response to Tam treatment. The evidence of the potential clinical relevance supports a role for NUPR1 in breast cancer progression, however, there is no significant association between NUPR1 and breast cancer subtypes^15^. In addition, *NUPR1*-depleted breast cancer cells show defects in autophagic degradation, resulting in premature senescence and reduced malignancy in vitro and in vivo. Our study also suggests that in the presence of autolysosomal degradation defects, enhancing autophagic flux may exacerbate the accumulation of nonfunctional autolysosomes, at least to some degree, leading to detrimental interventions. Taken together, these findings demonstrate that NUPR1 is a novel participant in the development of Tam resistance that maintains breast cancer cells at an elevated autolysosomal state through ESR1-mediated transcription.

As an ultimate attempt to preserve homeostasis, cancer cells can co-opt preexisting or treatment-induced signaling networks of epigenetic regulation as a unifying component of treatment failure to survive anticancer therapy^27^. It is generally accepted that whether autophagy is beneficial or detrimental is dependent upon the rate of induction and the appropriateness of the duration^28^. Modulation of autophagy in breast cancer has different and even opposing effects, indicating the need for a yet-to-be identified strategy when attempting to manipulate the autolysosomal process in the context of cancer therapy^29^. In the setting of *NUPR1*-depleted lung cancer cells, the imbalance between the increased on-rate of autophagic flux and the decreased off-rate of autolysosomal efflux impairs the autolysosomal process and results in premature senescence^13^. At present, we found that the level of NUPR1 is higher in a subpopulation of endocrine therapy resistant breast cancer cells than that in parental breast cancer cells, presumably through the elevated autophagic process induced by NUPR1-mediated transcription regulation. Furthermore, we noticed that *NUPR1* depletion in TamR cells severely abolished their survival upon Tam treatment. This outcome is due to a direct interaction between NUPR1 and ESR1, demonstrating that in addition to its ability to interact with transcription factors, NUPR1 can also interact with and modulate the activity of epigenetic modulators^16^. Although the precise mechanisms underlying the regulation of this process need to be investigated further, our data showed that NUPR1 is a promising druggable target against tumor resistance. Indeed, pharmacological inhibitors of autophagy as well as genetic interventions targeting various components of the autophagy machinery generally accelerate the demise of cells experiencing perturbations in homeostasis^30,31^. Very recently, transcriptional modulation by the CRISPR/deficient Cas9 system through trans-epigenetic remodeling has been tested in mouse models of diabetes, muscular dystrophy, and acute kidney disease^32^. This type of endogenous transcription regulation may pave new avenues for the development of targeted drug resistance to cancer therapies.

Tumor resistance can be defined as the selection of resistant clones and the acquired homeostatic resistance, but these two mechanisms are often indistinguishable because of tumor heterogeneity^33^. As a regulatory signaling pathway, the autophagic process is controlled through a myriad of signals, including transcriptional regulation, which may be more complicated than previously expected. NUPR1 is a transcriptional coregulator strongly induced by cellular stresses, and has the ability to mediate both tumor suppression and tumor development, presumably through its unique role in distinct transcriptional complexes^11,12^. Since *BECN1* is monoallelically deleted in 40–75% of cases of sporadic human breast, ovarian, and prostate cancers^25^, it is worth-noting that decreased mRNA levels of *BECN1* may contribute to the pathogenesis and progression of ESR1-negative breast cancers^34^. BECN1 is highly conserved and plays a key role in the initiation of autophagosome formation through binding of the apoptosis inhibitor BCL2, whereas disruption of the association between BECN1 and BCL2 induces the initiation of autophagy by freeing BECN1 to bind to Class III phosphatidylinositol 3-kinases (PI3Ks)^35^. Thus, the documented tumor suppressive effect of BECN1 on breast cancer cells may be partly due to its capacity to interact with Class III PI3Ks or HER2 (Erb-b2 receptor tyrosine kinase 2)^36^. Likely, the tumor-suppressive role of autophagy shows its ability to limit the accumulation of potentially oncoproteins, thereby preserving the intracellular homeostasis^37^. Upon Tam treatment, NUPR1 expression was increased, which is most likely due to adaptation of elevated autophagy to protect cells from cell death. Thus, impairment of NUPR1-mediated transcription control activates premature senescence, at least in part, via enhanced *BECN1* transcription and decreased IL-6 secretion.

Our data indicate that *NUPR1* depletion in ESR1-positive breast cancer cells overcomes Tam resistance; however, we do not know the exact functional associations of this protein except for those with ESR1. Likewise, ESR1, GREB, and PGR are functionally linked via alteration in the transcription of target genes that induce drug resistance programming^38,39^. Upon *NUPR1* depletion, proteins such as CDKN2A, CDKN1A, and CDKN1B, which directly regulate cell cycle progression, are involved in the NUPR1-mediated autolysosomal process, which is consistent with our previous observation in lung cancer cells^13^. More importantly, premature senescence, an irreversible state of cell cycle arrest, is also induced by *NUPR1* depletion in endocrine therapy resistant cells. Senescence is now increasingly considered an integral and widespread component that is potentially important for tumor development, tumor suppression and therapeutic response^40^. It is also broadly documented that premature senescence associated with distinctive increase in SA-β-gal activity, cell cycle arrest and induction of senescence-specific markers including cell cycle inhibitors CDKN1A/p21, CDKN1B/p27, and CDKN2A/p16^41,42^. However, the precise regulatory mechanisms of premature senescence in cancer cells are still largely unclear and further investigations will be imperative to provide a tangible way for its precise intervention in clinical setting. Noteworthy, it has also been previously shown that senescence-associated secretory phenotype (SASP) triggers the expression of tumor-promoting cytokines, including interleukin-6 (IL-6), which is an autophagy-inducing signal in cancers^43^. Based on these findings, we suggest that the elevated NUPR1 protein level may provide a novel biomarker for Tam resistance in ESR1-positive breast cancer cells.

Since NUPR1 plays a critical role in metastasis and drug resistance of cancer cells, it is now considered as a prognostic factor of poor outcomes^44^. When autolysosomal degradation is congested in *NUPR1*-depleted endocrine therapy resistant cells, this processing is also impaired, leading to accumulated organelles and premature senescence. Targeting the NUPR1-mediated autophagic regulation may render ESR1-positive breast cancer cells more sensitive to antiestrogen therapy. Thus, the implication of a combination of Tam and small-molecule inhibitors of autolysosomal efflux pathways needs to be further defined to prevent breast cancer recurrence.

## Materials and methods

### Immunohistochemistry

Human breast cancer tissues used in this study were obtained from Shanghai Outdo Biotech (HBre-Duc150Sur-02, Shanghai, China). These tissues were invasive ductal carcinoma prior to any treatments such as chemotherapy or radiotherapy. Histological sections (5 µm thick) were mounted on poly-l-lysine-coated slides. Slides of paraffin-embedded were deparaffinized in xylene and rehydrated in graded alcohols. Sections were pretreated with citrate buffer (0.01 M citric acid, pH 6.0) for 20 min at 95 °C. Then, at room temperature they were immersed in PBS containing 3% H_2_O_2_ for 10 min. After treatment with exposing them to 10% normal goat serum in PBS for 30 min at room temperature, breast cancer samples from patients and mouse were incubated at 4 °C overnight with primary antibodies as follows: NUPR1(dilution 1:200), PCNA(dilution 1:5000). Then the sections were rinsed with PBS, incubated with biotinylated goat anti-mouse IgG for 30 min at room temperature and treated with 3,3′-diaminobenzidine chromogen for 5 min at room temperature. Finally, sections were counterstained with hematoxylin for 2 min. Images were obtained with a CCD camera (Coolsnap ES, Roper Scientific) using Metamorph software (Molecular Devices). At least 50 cells from more than ten fields were counted for statistical analysis. Semiquantitative evaluation of staining was scored by two independent pathologists as follows: score = percentage of malignant cells staining positive (0 < 10%; 1, 10–25%; 2, 25–50%; 3, >50%) × mean stain intensity (0–3) as previously defined^45^. Scores were compared with overall survival, defined as the time from date of diagnosis to death. The variables of patients included age, gender, histological examination, metastasis, and pathological grade in Supplementary Table S1.

### Reagents and antibodies

Chemical reagents including Lysotracker red DND-99 (Life Technologies, USA), 4,6-diamidino-2-phenylindole (DAPI), trehalose, torin 1, chloroquine (CQ), bafilomycin A1 (BafA1), and 4-OH-tamoxifen (Tam) were purchased from Sigma-Aldrich (St. Louis, MO, USA). The restriction enzymes used in these experiments were purchased from New England BioLabs, Inc. (Beverly, MA). Antibodies used in this work are listed in Supplementary Table S2.

### Cell culture and viral infection

MCF-10A cells were obtained from Lonza (Basal, Switzerland) and were used at passages five. HEK-293 (ATCC^®^CRL-1573) and human breast cancer cell lines (MCF-7 (ATCC^®^HTB-22), T-47D (ATCC^®^HTB-133), BT-474 (ATCC^®^HTB-20), and MDA-MB-231 (ATCC^®^HTB-26)) were obtained from American Type Culture Collection (ATCC, Manassas, VA, USA).The cells were grown in DMEM (Gibco) supplemented with 10% fetal bovine serum and 0.01 mg/ml human recombinant insulin (Sigma) at 37 °C in 5% CO2 as recommended. Tam resistance cell lines were generated by chronic low-dose treatment with Tam in the presence of ethanol (OH) as vehicle^46^. To maintain Tam resistance, 0.4 μM Tam was added into the culture medium. For lentiviral transduction, HEK-293 cells were cotransfected with the transfer constructs and the third-generation packaging plasmids pMD2.VSVG, pMDLg/pRRE, and pRSV-REV, and fresh supernatant was used for infection as described previously^47^. shRNAs sequences are provided in Supplementary Table S3.

### Immunoblot

Proteins were extracted using RIPA cell lysis buffer and then subjected to sonication followed by centrifugation to remove insoluble material. The protein content was measured using BCA protein assay kit (Thermo Scientific, Waltham, MA, USA). Total protein in 1× Laemmli buffer (Bio-Rad, Hercules, CA, USA) was resolved by SDS-PAGE and electrotransferred to a pure nitrocellulose membrane (Life Sciences) at 4 °C. Immunoblot analysis was performed with the indicated antibodies and visualized on Kodak X-ray film using the enhanced chemiluminescence (ECL) detection system (Thermo Scientific). ACTB was used as a reference protein for normalization. Antibodies used in this work are listed in Supplementary Table S2.

### Transmission electron microscopy

Cells were trypsinized, washed with 0.1 M phosphate-buffered saline (PBS) (pH 7.4), and fixed with a solution containing 3% glutaraldehyde/2% paraformaldehyde in 0.1 M PBS (pH 7.4) for 2 h at RT. After fixation, the cells were washed with 0.1 M PBS (pH 7.4) and postfixed with 1% buffered osmium tetroxide for 45 min at RT, and stained with 1% uranyl acetate. After dehydration in graded series ethanol, the cells were embedded in Epon 812 (Fluka) medium and were polymerized at 70 °C for 2 d. Ultrathin sections were cut on a Leica Ultracut microtome and stained with uranyl acetate and lead citrate in a Leica EM Stainer. Digital TEM images were acquired from thin sections using a JEM 1010 transmission electron microscope (JEOL, Peabody, MA) at an accelerating voltage of 80 kV equipped with AMT Imaging System (Advanced Microscopy Techniques, Danvers, MA).

### Fluorescence microscopy and confocal microscopy

Multiple breast cancer cells were infected with a lentivirus expressing mcherry-GFP-LC3 fusion protein. After induction of autophagy, samples were examined using an epifluorescent microscope (Olympus BX61). Measurement of GFP and mcherry fluorescence was performed using a microplate reader with excitation/emission at 488/509 nm and 584/607 nm, respectively. For confocal microscopy, 1 × 10^5^ cells were seeded on bottom glass coated with poly-lysine (MatTek Corp.) for 30 min at room temperature. The cells were fixed with 4% paraformaldehyde (PFA) for 15 min at room temperature and washed two times in PBS. Nuclear counterstaining was performed with 4′,6-diamidino-2-phenylindole (DAPI) in PBS for 10 min. The cells were washed with PBS and were examined using a Zeiss LSM510 laser scanning confocal microscope (Carl Zeiss, Jena, Germany).

### Invasion assay

Invasion assays were performed in trans-well inserts with 8 μm pores (BD Biosciences) coated with 20% growth-factor-reduced Matrigel. 2 × 10^5^ cells were seeded into the upper chamber/per well in serum-free medium, and the lower chambers filled with complete media as a chemo-attractant. The chambers were incubated for 24 h at 37 °C with 5% CO_2_. Migrated cells on the undersides of filter membrane were fixed in 4% paraformaldehyde and washed three times in PBS. Then, the migrated cells were stained with crystal violet and counted using light microscopy. Experiments were performed in triplicate.

### Clonogenic survival assay

Cells (2 × 10^3^) were resuspended in DMEM medium containing 10% FBS with 0.35% agarose and layered on top of 0.6% agarose in DMEM and maintained for 14 days. Cells were fixed with 4% paraformaldehyde for 15 min and stained with crystal violet (0.2%) for 5 min. Colonies with more than 50 cells were counted as previously defined^48^. Images were digitally captured.

### Cell viability assay

Measurement of cellular ATP levels were performed using Cell Titer-Glo^®^ Luminescent Cell Viability assay kit according to the vendor’s suggestion (Promega Corporation, G7570, Madison, WI, USA). Briefly, cells were cultured in 96-well plates at 37 °C and 5% CO_2_. After the plate and its contents were equilibrated at room temperature for approximately 30 min, a volume of CellTiter-Glo^®^ reagent was added equal to the volume of cell culture medium present in each well and mixed for 2 min. Then, the ATP content was measured. The luminescence of each sample was normalized with the protein content.

### FACS

MCF-7 TAMR cells (1 × 10^5^) infected with Con shRNA or *NUPR1* shRNA were seeded in 6-well cell culture plates in 2.5 ml cell culture medium, with or without 0.4 μM Tam treatment, respectively. After 24 h, the cells were detached with Trypsin, stained with propidium iodide (PI) and measured with a FACS-Calibur (BD, Heidelberg, Germany). The percentage of PI positive cells was determined for each group.

### GLB1 staining

The cells were stained after fixation in 4% formaldehyde for 10 min with freshly prepared SA-β-galactosidase (SA-β-gal) staining solution overnight at 37 °C. GLB1 staining was performed using a Senescence β-Galactosidase Staining Kit (Cell Signaling Technology, #9860, Danvers, MA, USA) according to the manufacturer’s protocol. The number of GLB1-positive cells in randomly-selected fields was expressed as a percentage of all cells counted.

### Immunopurification and mass spectrometry

Cellular lysates from MCF-7TamR cells stably expressing FLAG-NUPR1 were applied to an equilibrated Anti-FLAG M2 affinity beads no more than 3 h at 4 °C. After binding, the beads were washed and the protein complex was eluted with FLAG peptide (#F3290, Sigma-Aldrich, St Louis, MO, USA). The proteins were resolved on SDS-PAGE and visualized by silver staining (Pierce Silver Stain Kit, #24612, Thermo Fisher Scientific, Waltham, MA), and subjected to LC-MS/MS sequencing and data analysis. The detailed information of NUPR1 binding proteins is listed in Supplementary Table S4.

### Immunoprecipitation

Cellular extracts were incubated with appropriate primary antibodies or normal rabbit/mouse immunoglobin G (IgG) at 4 °C overnight, followed by addition of protein A/G Sepharose CL-4B beads for 2 h at 4 °C. Beads were then washed and the immune complexes were subjected to SDS-PAGE followed by Western Blotting with corresponding antibodies.

### Proximity ligation assay (PLA)

PLA was performed using reagents and directions supplied in the Duolink In Situ Red Starter Kit Mouse/Rabbit (DUO92101, Sigma-Aldrich, St Louis, MO, USA). Slides were finally mounted using Duolink II Mounting Medium with DAPI and imaged using the Zeiss Axiovert 200 M.

### RNA isolation and quantitative real-time PCR

RNA isolation, reverse transcription, and quantitative real-time PCR were performed as described previously^13^. For target validation, TaqMan probes (Applied Biosystems) were used in quantitative real-time PCR. Gene expression was analyzed as previously described^13^, and primers used for qPCR are listed in Supplementary Table S5.

### RNA-Seq

Expression profiling was performed with total RNA extracted from three independent sets of cultured control shRNA against fire fly luciferase and *NUPR1* shRNA MCF-7TamR cells. RNA-seq libraries were constructed using Thermo Revert Aid First Strand cDNA Synthesis Kit (Cat#K1622) and then sequenced on Illumina HiSeq 2500. The RNA-seq data of NUPR1 knockdown in MCF-7TamR cells was deposited at the Gene Expression Omnibus (GEO) database with an accession number GSE104050.

### ChIP and ChIP-re-ChIP

ChIP experiments were conducted according to a previous protocol^49^. ChIP-re-ChIP was performed in MCF-7TamR cells as described previously^50^. Briefly, protein-DNA complexes were eluted two times from primary immunoprecipitation (IP) in 20 mM DTT at 37 °C, 30 min/per elution, and diluted 1:50 in buffer (1% Triton X-100, 2 mM EDTA, 150 mM NaCl, 20 mM Tris-HCl, pH 8.1) followed by re-ChIP with second antibodies. The immunoprecipitated DNA was isolated and quantified by real-time PCR with SYBR Green using the ABI Prism 7900 system (Applied Biosystems). Primers used in this study are shown in Supplementary Table S5.

### Luciferase assay

DNA fragments upstream of *BECN1* promoter were amplified fromT-47D genomic DNA using primers listed in Supplementary Materials, Table S5. BECN1 promoter and its TGACC deletion generated by site-directed mutagenesis were inserted into the *Xho* I and the *Nco* I site of the polylinker region pGL3-basic. The indicated cells were transiently cotransfected either in triplicate or in duplicate with pRL-CMV Renilla luciferase reporter, which was used for normalization. Then, these cells were harvested and assayed for luciferase activity assay (Dual-Luciferase^TM^ Reporter Assay System, E1910, Promega Corporation, Madison, WI, USA), following the manufacturers’ instructions at the time of 48 h after transfection.

### EMSA

Nuclear proteins were purified from *NUPR1*-overexpressing MCF-7TamR cells using NucBuster protein extraction kit (Novagen). The DNA probe for EMSA was prepared by double-stranded oligonucleotides 5′-CTCGAACTCCTGACCTCACGTGATC-3′ and 5′-GATCACGTGAGGTCAGGAGTTCGAG-3′ were end-labeled with biotin. Experiments were triplicated as described previously^48^.

### IL-6 ELISA

ELISA was performed with an IL-6 enzyme-linked immunosorbent assay kit (BD Biosciences; KHC0062). Breast cancer cells were incubated in serum-free medium for 12 h and 24 h, respectively. Supernatant samples were harvested and IL-6 levels were measured according to the manufacturer’s instructions. Quantitative data were normalized for cell number and were presented as average concentrations in pg/ml.

### Tumor xenografts

All animal studies were approved by the Institutional Animal Care and Use Committee of Tianjin Medical University. All mice were supplemented with estrogen pellets (0.72 mg of 17β-estradiol 60-day release from Innovative Research of America). A total of 2 × 10^6^ cells of MCF-7TamR or T-47DTamR cells of shRNA control cells and NUPR1 shRNA cells were suspended in 100 µl of PBS/Matrigel (1:1) and inoculated into the right and left axillary mammary fat pads of 4–5-week-old virgin female severe combined immune deficiency (SCID) mice, respectively. Mice were also treated with Tam pellets implanted subcutaneously (5 mg slow release pellet, Innovative Research of America). Tumor diameters were measured using a caliper, and volumes were estimated according to the formula: volume (mm^3^) = (longer diameter × shorter diameter^2^)/2. Mice were monitored to check for the subcutaneous tumors or weight once a week.

### Generation of CRISPR/dCas9-KRAB cells

MCF-7TamR and T-47DTamR cells were stably transduced with a lentiviral vector expressing dCas9-KRAB from a promoter of EFS-NS and selected with 8 μg/mL blasticidin. Then, cells were transduced with a lentiviral vector expressing sgRNAs targeting *NUPR1* or irrelevant *lacZ* control and selected with 0.5 μg/mL puromycin. sgRNA sequence targeting *lacZ* was used as a negative control. sgRNAs sequences are listed in Supplementary Table S5.

### Statistical analysis

Results were reported as mean ± SD (standard deviation) unless otherwise noted. Correlation of the expression levels between NUPR1 and survival rates were determined with Kaplan–Meier analysis using Mantel-Cox log-rank and Mantel–Haenszel hazard ratio testing (GraphPad Prism). The association of immunocytochemical staining with clinico-pathological characteristics was analyzed using *X*^2^ test. Statistical software SPSS 18.0 was used to evaluate the data in this study and differences were considered to be statistically significance at *P* < 0.05.

## Supplementary information

Supplementary information

Fig. S1 NUPR1 is involved in Tam resistance.

Fig. S2 NUPR1 is involved in autophagic survival.

Fig. S3 NUPR1 depletion inhibits tumorigenesis.

Fig. S4 Inactivation of NUPR1 by CRISPR-dCas9-KRAB-mediated sgRNA.

Fig. S5 Transcriptome validation in TamR cells.
