# Supplementary material for: Transcriptional coregualtor NUPR1 maintains tamoxifen resistance in breast cancer cells
Source: Cell Death Dis. 2021 Feb 4;12(2):149. doi: 10.1038/s41419-021-03442-z (PMC7862277; doi:10.1038/s41419-021-03442-z)
Supplement: Supplementary file 1 — Supplementary information [file 41419_2021_3442_MOESM1_ESM.docx]

**Supplementary figure legends**

**Fig. S1** **NUPR1 is involved in Tam resistance. a** Cell viability assay of MCF-7 and MCF-7TamR cells with or without 0.4 µM Tam treatment for the indicated time, compared to the alcohol (OH) control treatment (*n* = 3). ** *P*  <  0.01. **b** MCF-7TamR cells were transfected with *NUPR1* shRNA and/or treated with 0.4 µM Tam. Immunoblots confirmed the knockdown efficiency of 3 shRNAs against human *NUPR1*, with fire fly luciferase as a negative control (con) and ACTB as an internal control (upper panel) and immunocytochemistry of NUPR1 (lower panel).

**Fig. S2 NUPR1 is involved in autophagic survival. a** Immunoblots of LC3B and SQSTM1 in TamR cells as indicated, with ACTB as a loading control, in MCF-7TamR and T-47DTamR cells, respectively. Upper panels, immunoblots confirming the knockdown efficiency of shRNAs targeting *ATG5* or *ATG7*. **b** Electron micrograph of MCF-7, *NUPR1*-depleted MCF-7TamR and shRNA con cells. Note that the swelled cytoplasmic vacuoles are dispersed through the cytoplasm in the *NUPR1*-depleted MCF-7TamR cells (arrows). N, nuclei. M, Mitochondria. **c** Immunoblot analysis of LC3B, SQSTM1, and ACTB expression in *NUPR1*-depleted MDA-MB-231 cells treated with torin 1 or trehalose as indicated. **d** Immunoblot of CTSD in *NUPR1*-depleted and control cells as indicated, with ACTB as a loading control. **e** MCF-7TamR and T-47DTamR cells were transduced with shRNA con and *NUPR1* shRNA as indicated. Western blots show expression of p70S6K (T389), p70S6K (S371), p4E-BP, MTOR, pMTOR (Ser2448), and ACTB in the presence or absence of 10 μM torin 1. **f** Immunoblot analysis of LC3B, SQSTM1, and ACTB expression in *NUPR1*-depleted MCF-7TamR cells with trehalose or Baf A1 or combined treatment as indicated.

**Fig. S3 *NUPR1* depletion inhibits tumorigenesis.** **a** Matrigel invasion assay of MCF-7TamR and T-47DTamR cells upon *NUPR1* depletion, compared to their corresponding parental cells and shRNA con cells. Scale bars, 20 µm. **b** Microphotographs of T-47DTamR tumors and MCF-7 parental tumors treated with *NUPR1* shRNA as collected as in Fig. 3D. Tumor weight of T-47DTamR cells transduced with *NUPR1* shRNA and shRNA con (left panels). *** *P* < 0.001 compared to control. **c** H&E staining and IHC staining for PCNA shows the morphology of MCF-7TamR tumor xenografts, respectively. Scale bars, 50 µm.

**Fig. S4 Inactivation of *NUPR1* by CRISPR-dCas9-KRAB-mediated sgRNA.** **a** Immunoblot of the indicated proteins in MCF-7TamR or T-47DTamR cells subjected to *NUPR1* inactivation via CRISPR-dCas9-KRAB-mediated sgRNA system and in their corresponding control cells. **b** Cytochemical staining for GLB1 activity was performed in *NUPR1*-depleted MCF-7TamR and T-47DTamR cells. The percentage of GLB1 positive cells is shown in the histogram (right panel). The mean ± SD of three independent replicates is shown.

**Fig. S5** **Transcriptome validation in TamR cells.** **a** and **b** Quantitative RT-PCR was performed to confirm transcriptional changes of the indicated genes identified in the RNA-seq data. RNA levels were normalized to those of *GAPDH* and were presented as the relative fold change compared to the levels in shRNA con samples from MCF-7TamR cells (**a**) and T-47DTamR cells (**b**). **c** IL-6 ELISA analysis of serum-free culture supernatants from MCF-7TamR cells upon *NUPR1* depletion compared to the parental cells treated as indicated.

**Supplemental Tables**

Supplementary Table S1. The clinico-pathological characteristics of breast cancer tissue microarray specimens.

Supplementary Table S2. Primary antibodies used in this study.

Supplementary Table S3. shRNA sequences used in this study.

Supplementary Table S4. Mass spectrometry analysis of NUPR1-containing protein complex.

Supplementary Table S5. Oligos used in this study.

**Supplementary Table S1. The clinico-pathalogical characteristics of breast cancer tissue microarray specimens.**

| TNM status | | | |
| --- | --- | --- | --- |
|  | NUPR1 score low | | NUPR1 score high |
| T1N0M0 | 18 | | 5 |
| T1N1M0 | 7 | | 1 |
| T1N2M0 | 2 | | 1 |
| T1N3M0 | 0 | | 0 |
| T2N0M0 | 27 | | 16 |
| T2N1M0 | 14 | | 8 |
| T2N2M0 | 12 | | 1 |
| T2N3M0 | 5 | | 3 |
| T3N0M0 | 2 | | 1 |
| T3N1M0 | 1 | | 3 |
| T3N2M0 | 2 | | 1 |
| T3N3M0 | 1 | | 2 |
| *P* = 0.2839 χ^2^= 12.0211 2-tailed Chi-square test | | | |
| Clinical stage | | | |
|  | NUPR1 score low | | NUPR1 score high |
| Ⅰ | 1 | | 0 |
| Ⅰ-Ⅱ | 0 | | 2 |
| Ⅱ | 54 | | 15 |
| Ⅱ-Ⅲ | 23 | | 14 |
| Ⅲ | 13 | | 11 |
| *P* = 0.0287, χ^2^= 10.8140 2-tailed Chi-square test | | | |
| Age | | | |
|  | NUPR1 score low | | NUPR1 score high |
| Age (years) ≤ 55 | 42 | | 18 |
| > 55 | 49 | | 24 |
| *P* = 0.0353 for Age, unpaired, 2-tailed Student’s T-test | | | |
| Histological classification | | | |
|  | NUPR1 score low | NUPR1 score high | |
| Invasive ductal carcinoma | 80 | 36 | |
| Mucinous carcinoma | 0 | 1 | |
| Invasive lobular carcinoma | 1 | 0 | |
| Invasive ductal carcinoma with invasive micropapillary carcinoma | 3 | 3 | |
| Invasive ductal carcinoma with medullary carcinoma | 1 | 1 | |
| Invasive ductal carcinoma with invasive lobular carcinoma | 3 | 0 | |
| Invasive ductal carcinoma with mucinous carcinoma | 3 | 2 | |
| *P*=0.4952, χ^2^=5.3876, 2-tailed Chi-square test | | | |
| IHC status | | | |
|  | NUPR1 score low | NUPR1 score high | |
| ER negative | 38 | 14 | |
| ER positive | 53 | 28 | |
| *P*=0.3547, χ^2^=0.8566, 2-tailed Chi-square test | | | |
|  | NUPR1 score low | NUPR1 score high | |
| PR negative | 69 | 24 | |
| PR positive | 22 | 18 | |
| *P*=0.0290, χ^2^=4.7691, 2-tailed Chi-square test | | | |
|  | NUPR1 score low | NUPR1 score high | |
| HER2 negative | 38 | 14 | |
| HER2 positive | 53 | 28 | |
| *P*=0.3547, χ^2^=0.8566, 2-tailed Chi-square test | | | |

**Supplementary Table S2. Primary antibodies used in this study.**

| Primary Antibody | Dilution Ratio | Supplier |
| --- | --- | --- |
| ACTB (mouse monoclonal anti-) | 1:5,000 | Millipore (MAB1501) |
| ATG5 (rabbit polyclonal anti-) | 1:5,000 | Cell Signaling Technology (#2630) |
| ATG7 (rabbit monoclonal anti-) | 1:5,000 | Abcam (ab52472) |
| BECN1 (rabbit polyclonal anti-) | 1:5,000 | Abcam (ab55878) |
| Cathepsin D (mouse monoclonal anti-) | 1:2,000 | Santa Cruz Biotechnology(sc-377299) |
| CDK4 (rabbit monoclonal anti-) | 1:2,000 | Cell Signaling Technology (#9932) |
| CDK6 (mouse monoclonal anti-) | 1:2,000 | Cell Signaling Technology (#9932) |
| CDKN1A/p21Waf1 (rabbit monoclonal anti-) | 1:2,000 | Cell Signaling Technology (#9932) |
| CDKN1B/p27Kip1 (rabbit monoclonal anti-) | 1:2,000 | Cell Signaling Technology (#9932) |
| CDKN2A/p16INK4a (rabbit monoclonal anti-) | 1:2,000 | Abcam (ab108349) |
| CDKN2C/p18INK4C (mouse monoclonal anti-) | 1:1,000 | Cell Signaling Technology (#9932) |
| ESR1 (rabbit monoclonal anti-) | 1:5,000 | Abcam (ab108398) |
| FLAG (mouse monoclonal anti-) | 1:4,000 | Sigma Aldrich (F3165) |
| LC3B (rabbit polyclonal anti-) | 1:2,000 | Sigma Aldrich (L7543) |
| mTOR (rabbit monoclonal anti-) | 1:2,000 | Cell Signaling Technology (#2983) |
| NUPR1 (mouse monoclonal anti-) | 1:1,000 | Abcam (ab87454) |
| PCNA (rabbit polyclonal) | 1:1,000 | Abcam (ab18197) |
| Phospho-4E-BP (Thr37/46) (rabbit monoclonal anti-) | 1:1,000 | Cell Signaling Technology (#2855) |
| Phospho-mTOR S2448 (rabbit monoclonal anti-) | 1:2,000 | Cell Signaling Technology (#5536) |
| Phospho-p70S6K (Thr389) (rabbit monoclonal anti-) | 1:1,000 | Cell Signaling Technology (#9234) |
| Phospho-p70S6K (Ser371) (rabbit polyclonal anti-) | 1:1,000 | Cell Signaling Technology (#9208) |
| SQSTM1/p62 (rabbit polyclonal anti-) | 1:1,000 | Cell Signaling Technology (#7695) |

**Supplementary Table S3. shRNA sequences used in this study.**

| shRNA target sequence (5’-3’) | Efficiency (%) |
| --- | --- |
| *ATG5* (human, NM_00449) |  |
| #1 GCAACTCTGGATGGGATTG | 70 |
| #2 AGTGAACATCTGAGCTACC | 80 |
|  |  |
| *ATG7* (human, NM_001136031.2) |  |
| #1 AGCATCATCTTCGAAGTGA | 75 |
| #2 AAGAGAAAGCTGGTCATCA | 85 |
|  |  |
| *ESR1* (human, NM_000125.3) |  |
| #1 TCAAGGACATAACGACTAT | 85 |
|  |  |
| *NUPR1* (human, NM_012385) |  |
| #1 AGAGGAAACTGGTGACCAAGC | 50 |
| #2 GGAGGACCCAGGACAGGATCC | 80 |
| #3 GGATGAATCTGACCTCTATAG | 90 |
|  |  |
| *Fire fly luciferase* (con) |  |
| CGTACGCGGAATACTTCGATT | Irrelevant control |

| Band (kDa) | Name | Accession # | Description | Peptides |
| --- | --- | --- | --- | --- |
| 8.9 | FLAG-  NUPR1 | O60356 | Nuclear protein 1 | EAAANTNRPSPGGHER  KLVTKLQNSER |
| 140.9 | DHX9 | Q08211 | ATP-dependent RNA helicase A | LAAQSCALSLVR  ELDALDANDELTPLGR  DVVQAYPEVR |
| 94.3 | HSPA4 | P34932 | Heat shock 70 kDa protein 4 | VLATAFDTTLGGR  ELSTTLNADEAVTR  GCALQCAILSPAFK |
| 66.2 | ESR1 | P03372 | Estrogen receptor alpha | LASTNDKGSMAMESAK  SIQGHNDYMCPATNQCTIDK  AGLTLQQQHQR |
| 72.3 | HSPA5 | P11021 | 78 kDa glucose-regulated protein | KSDIDEIVLVGGSTR  VLEDSDLKKSDIDEIVLVGGSTR  KKELEEIVQPIISK  TKPYIQVDIGGGQTK |
| 70.9 | HSPA8 | P11142 | Heat shock cognate 71 kDa protein | LDKSQIHDIVLVGGSTR  IINEPTAAAIAYGLDKK  STAGDTHLGGEDFDNR |
| 73.6 | HSPA9 | P38646 | Stress-70 protein | VINEPTAAALAYGLDK  QAVTNPNNTFYATK  SDIGEVILVGGMTR |
| 38.6 | TRIM21 | F5H012 | E3 ubiquitin-protein ligase TRIM21 | LGDTQQSIPGNEER  QLQELEKDEREQLR  IHAEFVQQK  QLQELEKDER |
| 38.6 | ANXA2 | P07355 | Annexin A2 | TNQELQEINR  TPAQYDASELK  GVDEVTIVNILTNR |
| 35.9 | YBX1 | P67809 | Nuclease-sensitive element-binding protein 1 | GAEAANVTGPGGVPVQGSK  NDTKEDVFVHQTAIKK |
| 24.8 | SYPL1 | C9JYN0 | Synaptophysin-like protein 1 | TVTATFGYPFR |
| 11.5 | LYZ | F8VV32 | Lysozyme C | STDYGIFQINSR |
| 14.3 | ACP1 | P24666-3 | Isoform 3 of Low molecular weight phosphotyrosine protein phosphatase | SPIAEAVFR  IELLGSYDPQK |
| 13.2 | S100A9 | P06702 | Protein S100-A9 | LGHPDTLNQGEFK  NIETIINTFHQYSVK |

**Supplementary Table S4. Mass spectrometry analysis of NUPR1-containing protein complex.**

**Supplementary Table S5. Oligos used in this study.**

| **List of genes and the primer sequences used for qRT-PCR** | | |
| --- | --- | --- |
| Gene name | Sequence (Forward/Reverse, 5’-3’) | |
| *ATG5* (NM_004849.2) | | GTGAGATATGGTTTGAATATGAAGGC/CTCTTAAAATGTACTGTGATGTTCCAA |
| *ATG7* (NM_006395.2) | AGGAGATTCAACCAGAGACC/GCACAAGCCCAAGAGAGG | |
| *ATG9B* (NM_173681) | TCTTCCAGCTGGGACAATTTAT/CAGGTCTGGTATGGTTACTTGG | |
| *ATP6V0E1* (NM_003945.3) | TCTTGGTGCCTTGGTTCAT/GTCCAAAGAGAGGGTTGAGTT | |
| *ATP6V0A4* (NM_130840.2) | CCCACGGGAAATGATTA/GGTTTCCGTCTCAAAGA | |
| *ATP6V0D2* (NM_152565.1) | GTCTTACCTTGAGGCATTCT/GGTCTCTCGGTCTTCTTTG | |
| *BCL2* (NM_000633.2) | AGGGGGAAACACCAGAATCAAGTG/CCCAGAGAAAGAAGAGGAGTTATAA | |
| *BECN1* (NM_003766.3) | GGAGAGGAGCCATTTATTGAAACT/AGAGTGAAGCTGTTGGCACTTTCTG | |
| *CTSB* (NM_001908.4) | TCTGAGGAACATTGTAGGAAAGG/TCCCAAAGTGCTGGGATTAC | |
| *CTSD* (NM_001909.4) | CAACAGCGACAAGTCCAGC/CTGAATCAGCGGCACGGC | |
| *CTSL* (NM_001912.4) | CTGTGGATTGGAGAGAGAAAGG/CAAGAGCACCAGTAGCACTAAA | |
| *CYP1B1* (NM_000104.3) | GGTTCCTGTTGACGTCTTG/AGCACCGACAGGAGTAG | |
| *ERN1* (NM_001433.3) | CCATCAACCTCTCTTCTGTATC/GGCCGCATAGTCAAAGTAG | |
| *ESR1* (NM_000125.3) | CCACCAACCAGTGCACCATT/GGTCTTTTCGTATCCACCTTTC | |
| *GATA4* (NM_001308093.1) | GAAGCCCAAGAACCTGAATAAA/TGTGCCCGTAGTGAGATG | |
| *GREB1* (NM_014668.3) | CTTCACGGAATTCTCCAATCA/GCTGGGAAGAGGGAACT | |
| *IL6* (NM_000600.4) | GTGAAAGCAGCAAAGAG/TCCTCACTACTCTCAAATC | |
| *LAMP1* (NM_005561.3) | CAAGAGTGGCCCTAAGAACAT/GTGAGTGTATGTCCTCTTCCAAA | |
| *LAMP2* (NM_001122606.1) | CTGTGACATATAATGGAAGC/TCAGGAAATGTTGTGTTATC | |
| *LAMP3* (NM_014398.3) | TCACCTCGGAGATACTT/TCCCACTTCACTGATATAAT | |
| *LCN2* (NM_005564) | AGCAGAACTTCCAGGACAAC/TTGCGGGTCTTTGTCTTCTC | |
| *MAP1LC3B* (NM_022818.4) | CGGTGATAATAGAACGATACAAGG/CTGAGATTGGTGTGGAGACG | |
| *mTOR* (NM_004958.3) | TTCTGGTGCGACACCGAATC/CATCGGGTTGTAGGCCTGTG | |
| *NEDD9* (NM_001142393.1) | ACGAGTACCCATCCAGATAC/TCTCCCACTGGAACTGAAA | |
| *NUPR1* (NM_012385) | CCTGGATGAATCTGACCTCTA/TCGCTTCTTCCTCTCTGAA | |
| *PGR* (NM_001202474.3) | CTGACACCTCCAGTTCTTTG/CCCACTGACGTGTTTGTAG | |
| *PTEN* (NM_000314.4) | TGGCTAAGTGAAGATGACAATCATG/GCACATATCATTACACCAGTTCGT | |
| *RAB31* (NM_006868.3) | CACAACATCAGCCCTACTATT/CATGGGAGCCAATGAATGA | |
| *SIAH2* (NM_005067.5) | CACAGCCATAGCACATCTT/AATTGTGGGTCCTGACTTG | |
| *SPTLC3* (NM_018327.2) | TGGTCTTGCAGCCAAGTATG/CCTCCAACTCCTTGTGCTTATC | |
| *SQSTM1* (NM_003900.4) | AATGGGTCCACCAGGAAAC/CGATGGACCAGAAGCTGATT | |
| *TET2* (NM_001127208.2) | CTGGCTGACAAACTCTACTC/TGCTTCTGGCAAACTTACA | |
| *TFEB* (NM_001271944.1) | CGCATCAAGGAGTTGGGAAT/CTCCAGGCGGCGAGAGT | |
| *GAPDH* (NM_002046) | GTGGACCTGACCTGCCGTCT/GGAGGAGTGGGTGTCGCTGT | |
| **Primers used in ChIP assays** | | |
| Primer Name | Primer (5’-3’) | |
| *BECN1*-1F | TGGGTTAAGCAGTGGTTTC | |
| *BECN1*-1R | TGCCCAACTATCACCATAATC | |
| *BECN1*-2F | GGGTTTGAGATGGAGTTTCC | |
| *BECN1*-2R | TGGTTAAATCCCGTCTCTACTA | |
| *BECN1*-3F | CTCCCAAAGTGCTGGAATTA | |
| *BECN1*-3R | GCAGGAGAATCGCTTGAA | |
| *BECN1*-4F | CTCAGCCTCCCGAGTAG | |
| *BECN1*-4R | GCTCACGCCTGTAATCTC | |
| *BECN1*-5F | CTGGTCTCGAACTCCTGA | |
| *BECN1*-5R | AGCGGATGAACAAGGTTAC | |
| *BECN1*-6F | CTTAGAAGGGAGTCTGGGAA | |
| *BECN1*-6R | ATGGTAAAGGGAGGGAAGT | |
| *BECN1*-7F | CTGGACACGAGTTTCAAGAT3 | |
| *BECN1*-7R | ACAGGGAGCCAGATGAA | |
| *CYP1B1*-1F | CCTGTTTGGTGGTCTCTTC | |
| *CYP1B1*-1R | TTGTAGGATGCACCCTTATTT | |
| *CYP1B1*-2F | GCCTCCCTCATAAGCAATTTA | |
| *CYP1B1*-2R | CTGGGCAGCAGAGTTTAAG | |
| *CYP1B1*-3F | AGTCACGCAACCTCTCT | |
| *CYP1B1*-3R | TGTACTGGCTTCATCAAAGG | |
| *CYP1B1*-4F | TGACTGGAGCCGACTTT | |
| *CYP1B1*-4R | GCTCCGGCGAACTTTATC | |
| *CYP1B1*-5F | TGAAAGCCTGCTGGTAGA | |
| *CYP1B1*-5R | TCCCACTCCAGAGTCAAAG | |
| *CYP1B1*-6F | GTGAGGAAACCTCGACTTTG | |
| *CYP1B1*-6R | CCCTCACTGGAAGCTTTAAC | |
| *GREB1*-1F | GGATGTTCTGGACTTGCTATT | |
| *GREB1*-1R | GCTCCTTTATGCCCAGTTT | |
| *GREB1*-2F | CTGACTCGCCAGAAGAAATAA | |
| *GREB1*-2R | CCATGGAAGGTAAACGGATT | |
| *GREB1*-3F | TGCAGACACTATAGTTCCATAA | |
| *GREB1*-3R | GCAATATAAATGTGTCACAACG | |
| *GREB1*-4F | CTCTGACGTGTCACTCTTAAA | |
| *GREB1*-4R | ACAAAGTGGCAATCCTAGTC | |
| *GREB1*-5F | CAGGGAGATATTGTGATCAGG | |
| *GREB1*-5R | GTGCAGGCTTGTAGAGATT | |
| *GREB1*-6F | TCTGTAGCAGCAACGAATG | |
| *GREB1*-6R | CCTCAGAGGTACAGGTAGAA | |
| *GREB1*-7F | AAGTTGCTCAGCCAAGTC | |
| *GREB1*-7R | CCCAGGCAGCTTCTTTC | |
| *NEDD9*-1F | TGAGTGGCTGGATTAGGT | |
| *NEDD9*-1R | CTGGGCACTGGCATAAAT | |
| *NEDD9*-2F | CTGCTGTCCTTGAGTGTTT | |
| *NEDD9*-2R | TGGTAGGGAGCTTGGATT | |
| *NEDD9*-3F | GATTGCAGTGAGCCGATATT | |
| *NEDD9*-3R | GGAAAGCACAAGGGTGTT | |
| *NEDD9*-4F | CCTGCAAACTAACGACTCTC | |
| *NEDD9*-4R | GCCTTGCTTCTCCTCATTT | |
| *NEDD9*-5F | GAATTGTCTTCCAGCTCCTT | |
| *NEDD9*-5R | CTCTCTCTCTCTGTCCCTTT | |
| *NEDD9*-6F | CCACCGTCTGATAAAGCAA | |
| *NEDD9*-6R | GCTAGTGGGACAAGGTAATG | |
| *RAB31*-1F | GGATGTTCTGGACTTGCTATT | |
| *RAB31*-1R | GCTCCTTTATGCCCAGTTT | |
| *RAB31*-2F | CTGACTCGCCAGAAGAAATAA | |
| *RAB31*-2R | CCATGGAAGGTAAACGGATT | |
| *RAB31*-3F | TGCAGACACTATAGTTCCATAA | |
| *RAB31*-3R | GCAATATAAATGTGTCACAACG | |
| *RAB31*-4F | CTCTGACGTGTCACTCTTAAA | |
| *RAB31*-4R | ACAAAGTGGCAATCCTAGTC | |
| *RAB31*-5F | CAGGGAGATATTGTGATCAGG | |
| *RAB31*-5R | GTGCAGGCTTGTAGAGATT | |
| *RAB31*-6F | TCTGTAGCAGCAACGAATG | |
| *RAB31*-6R | CCTCAGAGGTACAGGTAGAA | |
| *RAB31*-7F | AAGTTGCTCAGCCAAGTC | |
| *RAB31*-7R | CCCAGGCAGCTTCTTTC | |
| *GAPDH*-F | GCCCTAATTATCAGGTCCAGGCTAC | |
| *GAPDH*-R | CTGGGGGCCTGGAGAGGAAGG | |
| **Primers used to generate *BECN1* promoter-luciferase reporters** | | |
| Primer Name | Primer (5’-3’) | |
| *BECN1*-1547-forward | TCCAGCCTGGGTAACAGAG | |
| *BECN1*-992-forward | CTCTGCTAATCCCCAAACAG | |
| *BECN1*-501-forward | GTCGCCCAGGCTGGAGT | |
| *BECN1*-153-forward | TTGTTCATCCGCTGAAGCC | |
| *BECN1*+116-reverse | TGCTCTTCACCTCGGGAG | |
| **sgRNA sequences for *NUPR1*** |  | |
| Primer Name | Sequence (Forward/Reverse, 5’-3’) | |
| *sgNUPR1* #1 | ACCTGATCGAGGGCTTCACC/GGTGAAGCCCTCGATCAGGT | |
| *sgNUPR1* #2 | ATCATGGCCTCACCTGATCG/CGATCAGGTGAGGCCATGAT | |
| *sglacZ* con | CGATCGTAATCACCCGAGTG/CACTCGGGTGATTACGATCG | |
