# Supplementary figures and images for: Transcriptional coregualtor NUPR1 maintains tamoxifen resistance in breast cancer cells
Source: Cell Death Dis. 2021 Feb 4;12(2):149. doi: 10.1038/s41419-021-03442-z (PMC7862277; doi:10.1038/s41419-021-03442-z)

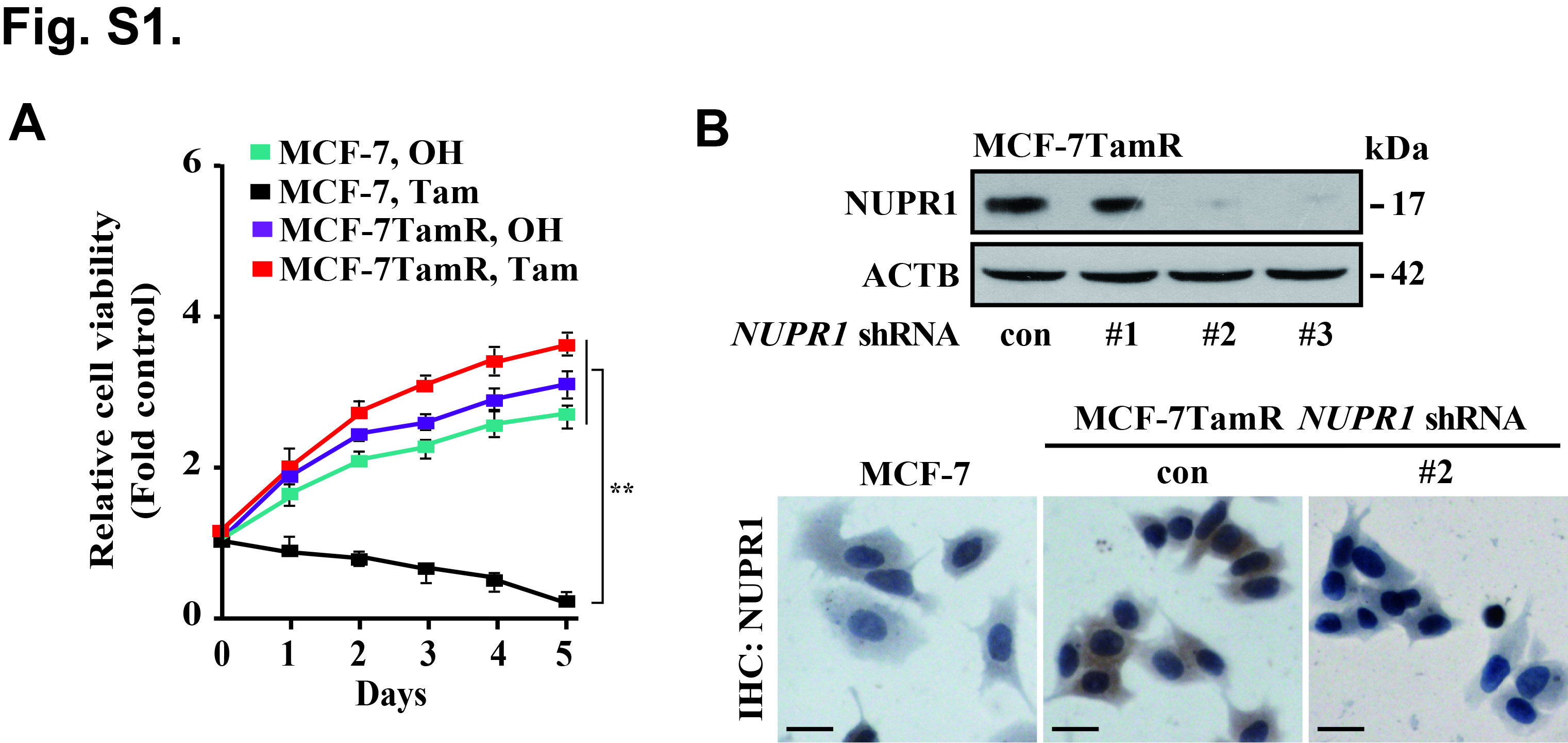

Supplement: Supplementary file 2 — Fig. S1 NUPR1 is involved in Tam resistance. [file 41419_2021_3442_MOESM2_ESM.tif]

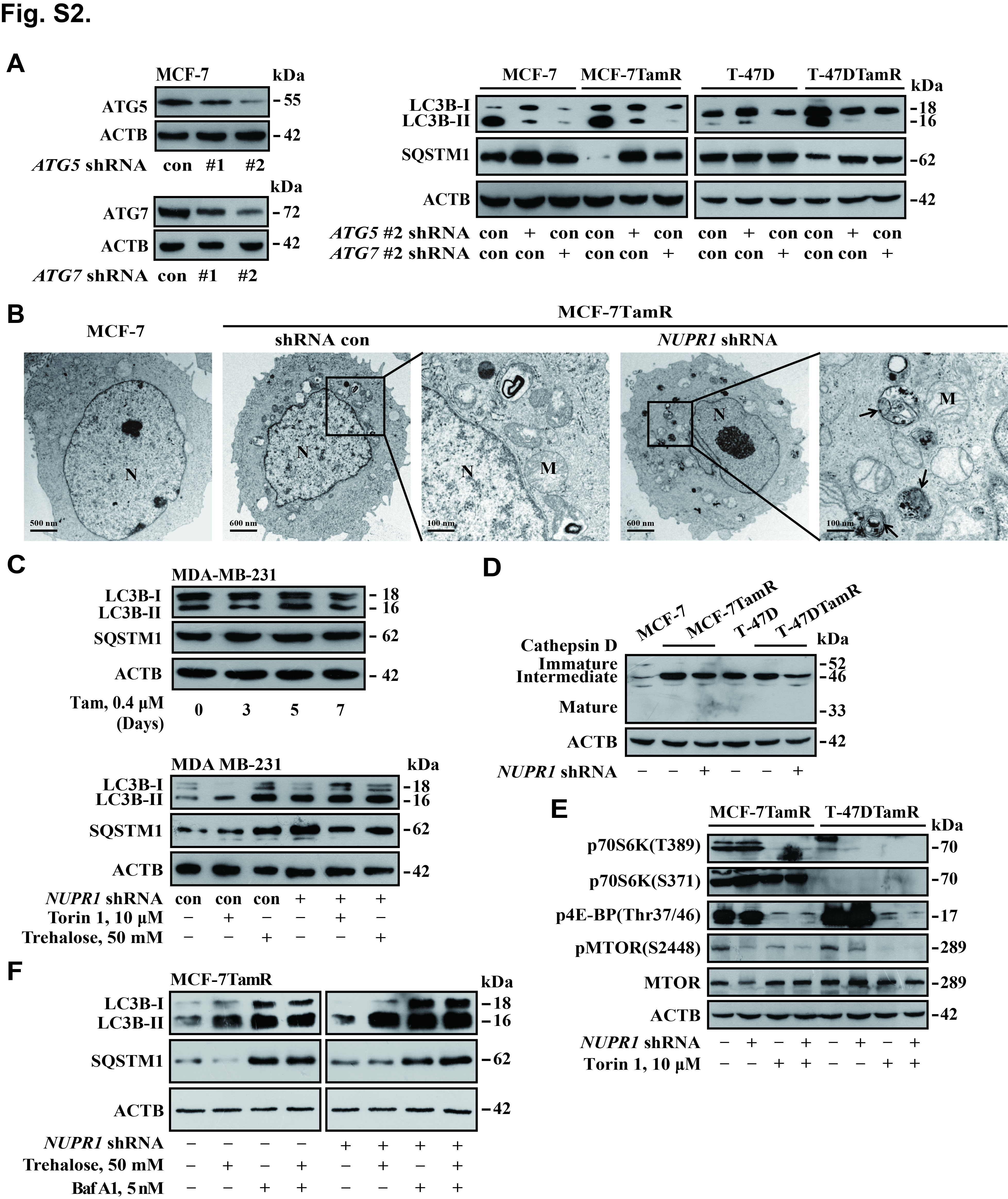

Supplement: Supplementary file 3 — Fig. S2 NUPR1 is involved in autophagic survival. [file 41419_2021_3442_MOESM3_ESM.tif]

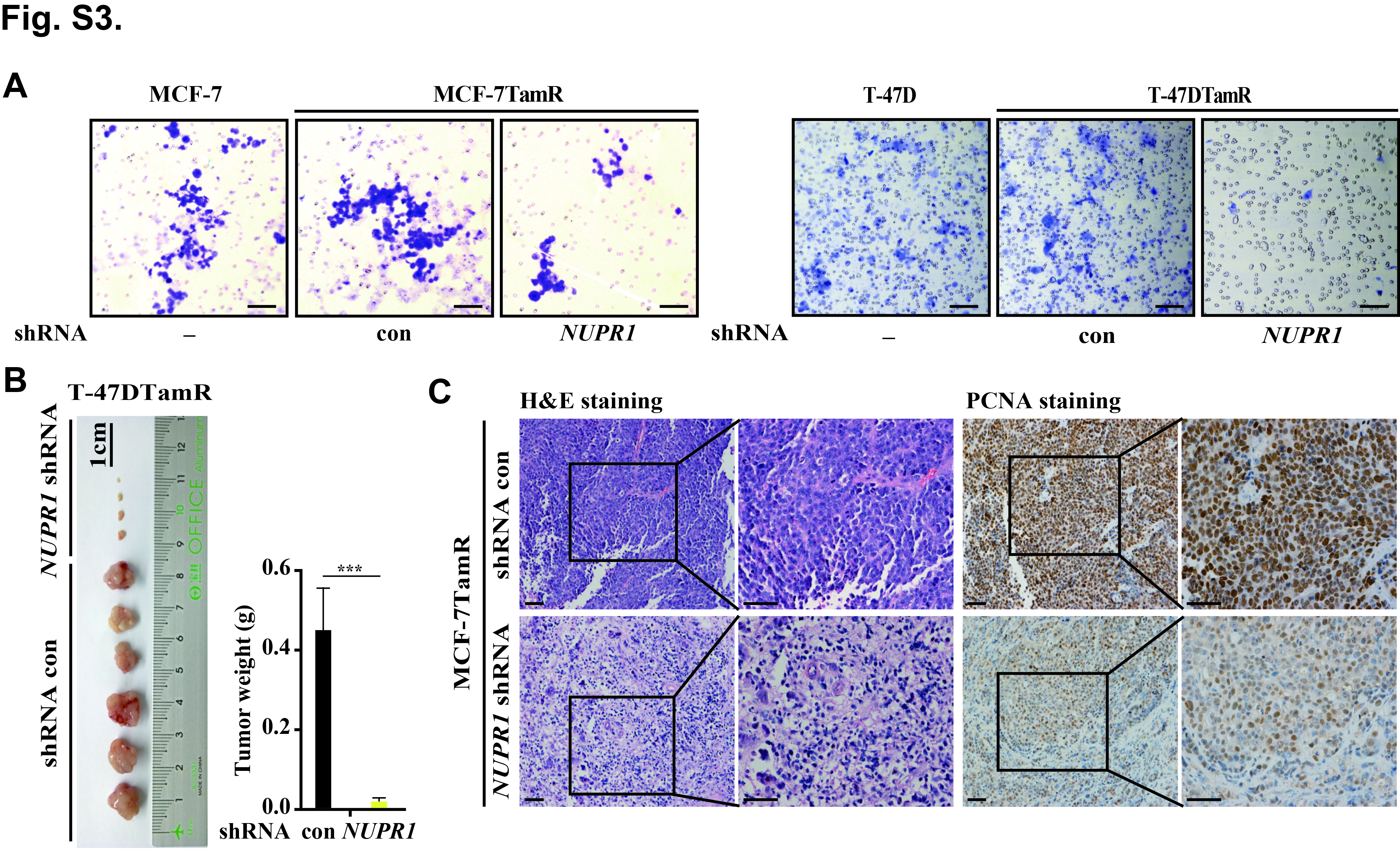

Supplement: Supplementary file 4 — Fig. S3 NUPR1 depletion inhibits tumorigenesis. [file 41419_2021_3442_MOESM4_ESM.tif]

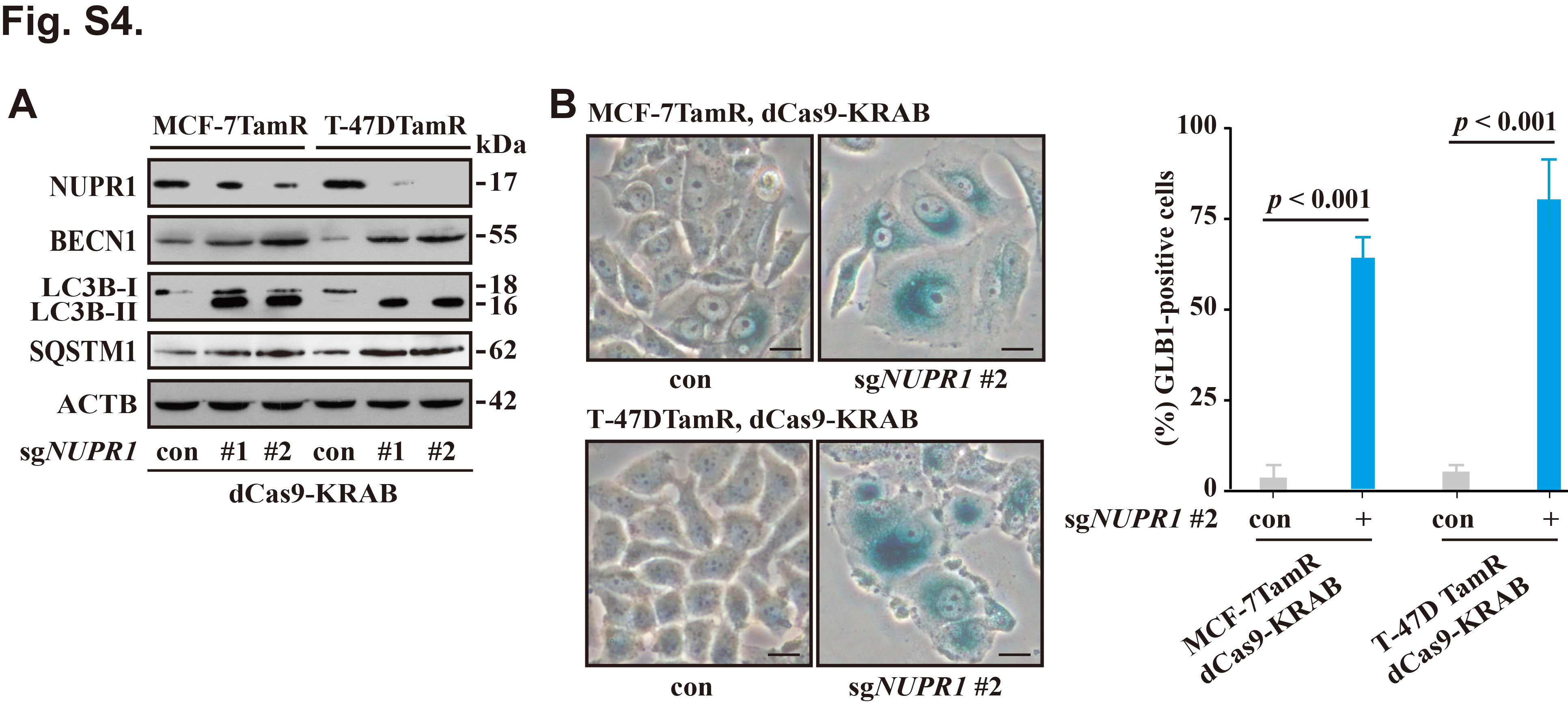

Supplement: Supplementary file 5 — Fig. S4 Inactivation of NUPR1 by CRISPR-dCas9-KRAB-mediated sgRNA. [file 41419_2021_3442_MOESM5_ESM.tif]

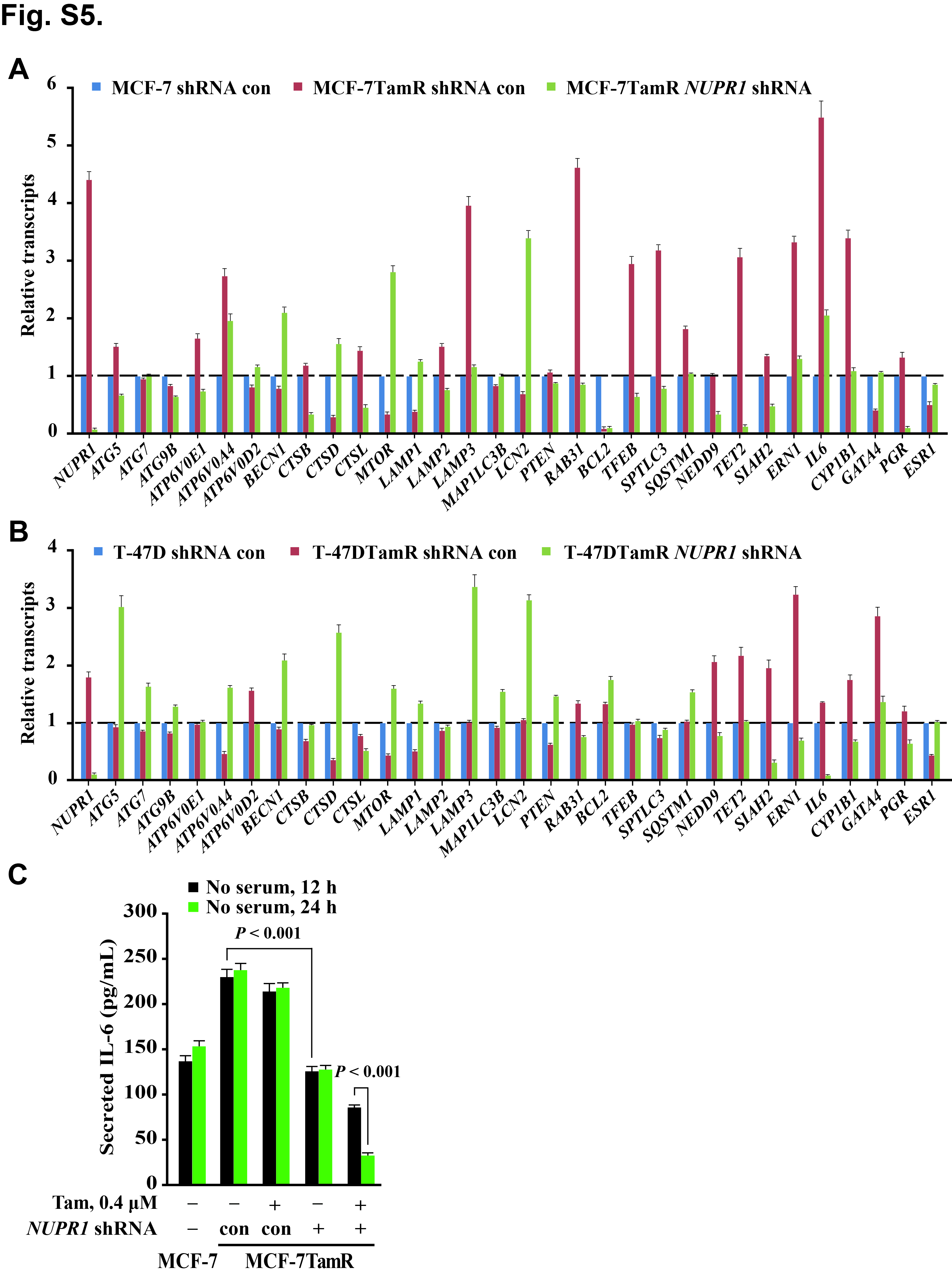

Supplement: Supplementary file 6 — Fig. S5 Transcriptome validation in TamR cells. [file 41419_2021_3442_MOESM6_ESM.tif]
